# CLPs-miR-103a-2-5p inhibits proliferation and promotes cell apoptosis in AML cells by targeting LILRB3 and Nrf2/HO-1 axis, regulating CD8 + T cell response

**DOI:** 10.1186/s12967-024-05070-5

**Published:** 2024-03-14

**Authors:** Qingyan Cen, Jianyu Chen, Jiaxin Guo, Mu Chen, Hao Wang, Suwan Wu, Honghao Zhang, Xiaoling Xie, Yuhua Li

**Affiliations:** Department of Hematology, Zhujiang Hospital, Southern Medical University, Guangzhou, 510282 Guangdong People’s Republic of China

**Keywords:** AML, LILRB3, microRNA-103a-2-5p, Nrf2/HO-1, CD8, Cationic liposomes, Immunology

## Abstract

**Background:**

LILRB3, a member of the leukocyte immunoglobulin-like receptor B (LILRB) family, has immunosuppressive functions and directly regulates cancer development, which indicates that LILRB3 is an attractive target for cancer diagnosis and therapy. Novel therapeutic treatments for acute myeloid leukemia (AML) are urgent and important, and RNA therapeutics including microRNAs (miRNAs) could be an effective option. Here, we investigate the role of dysregulated miRNA targeting LILRB3 in the AML microenvironment.

**Methods:**

Potential miRNAs binding to the 3ʹ-untranslated region (3ʹ-UTR) of the LILRB3 mRNA were predicted by bioinformatics websites. Then, we screened miRNAs targeting LILRB3 by quantitative real-time PCR, and the dual luciferase reporter assay. The expression of LILRB3 and microRNA (miR)-103a-2-5p in AML were determined and then their interactions were also analyzed. In vitro, the effects of miR-103a-2-5p were determined by CCK8, colony formation assay, and transwell assay, while cell apoptosis and cell cycle were analyzed by flow cytometry. Cationic liposomes (CLPs) were used for the delivery of miR-103a-2-5p in the AML mouse model, which was to validate the potential roles of miR-103a-2-5p in vivo.

**Results:**

LILRB3 was upregulated in AML cells while miR-103a-2-5p was dramatically downregulated. Thus, a negative correlation was found between them. MiR-103a-2-5p directly targeted LILRB3 in AML cells. Overexpressed miR-103a-2-5p significantly suppressed the mRNA and protein levels of LILRB3, thereby inhibiting AML cell growth and reducing CD8 + T cell apoptosis. In addition, overexpressed miR-103a-2-5p reduced both the relative expression of Nrf2/HO-1 pathway-related proteins and the ratio of GSH/ROS, leading to the excessive intracellular ROS that may promote AML cell apoptosis. In the mouse model, the delivery of miR-103a-2-5p through CLPs could inhibit tumor growth.

**Conclusions:**

MiR-103a-2-5p serves as a tumor suppressor that could inhibit AML cell proliferation and promote their apoptosis by downregulating LILRB3 expression, suppressing the Nrf2/HO-1 axis, and reducing the ratio of GSH/ROS. Besides, our findings indicate that miR-103a-2-5p may enhance the CD8 + T cell response by inhibiting LILRB3 expression. Therefore, the delivery of miR-103a-2-5p through CLPs could be useful for the treatment of AML.

**Supplementary Information:**

The online version contains supplementary material available at 10.1186/s12967-024-05070-5.

## Introduction

Acute myeloid leukemia (AML) is a malignant disease associated with the abnormal development of the myeloid hematopoietic stem/progenitor cells. According to the National Cancer Institute (NCI) statistics, there are almost 20,380 new instances of AML and 11,410 fatalities by 2023 [1]. Data shows that the incidence is about 2/100,000, in those under 65 while 20/100,000, in those over 65, indicating that the incidence gradually increases with the growth of age [2]. Although new molecular-targeted drugs, including FLT 3 inhibitors, IDH 1 inhibitors, IDH 2 inhibitors, and Bcl-2 inhibitors, have brought a new dawn to the treatment of AML patients [3, 4] as research on the molecular biology of diseases deepens, these patient’s prognosis is still poor. Thus, additional research on novel therapeutic targets and the pertinent mechanisms of AML is beneficial for the development of creative therapeutic approaches.

The LILRBs, a group of inhibitory leukocyte immunoglobulin-like receptors with the same immunoreceptor tyrosine-based inhibitory motif (IMTM) as immune checkpoint proteins such as CTLA 4, and PD-1, are novel immune checkpoint protein molecules [5]. LILRB3, a member of the LILRB family, might play two different functions in the biology of cancer [6], which could directly regulate cancer development and have immune inhibitory functions. Therefore, LILRB3 is an attractive diagnostic and therapeutic target for cancer [7, 8].

MicroRNAs (miRNAs) are a class of endogenous non-coding single-stranded RNA, made up of 18–23 nucleotides, which directly combine with 3’-UTRs of the target gene to induce gene degradation or regulate gene expression after transcription [9, 10]. Many studies have confirmed the strong relationship between miRNAs and the initiation, development, invasion, and metastasis of tumors. Therefore, the important role of miRNAs in tumor diagnosis and targeted therapy is gaining increasing attention [11]. As evidenced by earlier research, miRNAs are involved in the differentiation, proliferation, and survival of leukemic hematopoietic cells, affecting treatment response and prognosis, which suggests that miRNAs may be viable therapeutic targets for AML [12–14]. MiR-103a-2-5p, a tumor suppressor target, could inhibit the growth and induce apoptosis of tumor cells [15]. However, the important significance of miR-103a-2-5p in AML mentioned in this study remains undefined.

Nanoplatform-mediated chemotherapy drug delivery has contributed to the development of clinical cancer therapy [16]. Liposomes, preferred as widely used drug delivery systems, effectively protect nucleic acid molecules from inactivation and degradation [17]. Research has demonstrated that liposome-mediated gene transfer therapies have become the most promising means of gene transfer [18].

The purpose of this study is to explore the bioregulation of miR-103a-2-5p in acute leukemia cell lines. Furthermore, through the liposome-mediated miR-103a-2-5p transfer treatment, this study aims to establish a theoretical foundation for potential clinical application in the future.

## Materials and methods

### Ethics statement

This study was authorized and supervised by the Ethics Committee of the Zhujiang Hospital of Southern Medical University. Every participant endorsed an informed consent form. The Laboratory Animal Ethics Committee of the Zhujiang Hospital of Southern Medical University accepted all animal experiments involving nude mice. Considerable attempts were made to minimize both the number of animals and their suffering.

### Cell lines and culture conditions

AML cell lines (THP-1, MOLM-13, MV4-11, OCI-AML2, OCI-AML3, KG1a, HL-60, NB4, and U937), B-ALL cell lines (REH and NALM-6), CML cell (K562), the normal bone marrow cell line (HS-5), and the 293 T cells were maintained in our laboratory, the Zhujiang Hospital Hematological Laboratory. Before being used, cell lines were identified using short tandem repeat (STR) profiling and confirmed to be mycoplasma-free immediately. All tested cells were maintained at 37 ℃ in a 5% CO_2_ humidified environment in RPMI-1640 medium supplemented with 10% fetal bovine serum (FBS). HEK293T cells were cultured in Dulbecco’s modified Eagle’s medium (DMEM) under the same conditions. Ficoll-Hypaque density gradient was used to isolate bone marrow mononuclear cells (BMMCs) and peripheral blood mononuclear cells (PBMCs) from AML patients or healthy donors according to the standard protocols. The PBMCs were stimulated with anti-CD3 and anti-CD28, and cultured in RPMI 1640 medium in the presence of 10% FBS and interleukin-2 (IL-2, 50U/ml). For the detection of T cell apoptosis, AML cell lines (THP-1, OCI-AML3, OCI-AML2, MV4-11) were co-cultured at different rations with activated PBMCs. After 24 h or 72 h, cells were detected by FACS.

### Bioinformatics analysis

We used three distinct databases, including Target Scan (https://www.targetscan.org/), miRDB (https://mirdb.org/), and miRWalk (http://mirwalk.umm.uni-heidelberg.de/), to search for potential miRNAs regulating 3ʹ-UTR of LILRB3 mRNA. The predicted miRNAs by all three databases are shown in Additional file 1: Table S1. With the aid of quantitative reverse transcription polymerase chain reaction (qRT-PCR), the expression of overlapping miRNAs from all three databases was verified.

### Clinical sample collection

Using Ficoll-Hypaque density gradient centrifugation, bone marrow aspirates from AML patients and healthy donors were used to isolate BMMCs. With Ficoll-Hypaque density gradient centrifugation and the CD34 MicroBead kit (Miltenyi Biotec, Auburn, CA, USA), CD34 + PBMC were isolated from healthy human peripheral blood (PB) by following the manufacturer's instructions. Fluorescence-activated cell sorting (FACS) was used to confirm that the isolated cells were over 95% pure. Following informed consent from the patients, blood samples were taken in accordance with the 1983 revision of the Declaration of Helsinki. All studies involving human samples were approved by the Zhujiang Hospital Institutional Review Board. The detailed information on AML patients is shown in Additional file 2: Table S2. Bone marrow samples were stored at − 80 ℃ before RNA purification.

### Transfection

MiRNAs were overexpressed through the transfection of certain miRNA mimics. MiRNA mimics, together with their respective NC mimics (miR-NC), were produced by IGEbio (Guangdong, China). Cells were transfected for 48 h with the aid of Lipofectamine 3000 Reagent (Invitrogen, Shanghai, China).

### Plasmid construction and luciferase reporter assay

To construct the reporter vector LILRB3-WT, the full-length 3ʹ-UTR sequences (827 bp) of LILRB3 were cloned into pmirGLO Dual-luciferase vectors (IGEbio, China). TargetScan, a publicly available bioinformatics database, was utilized to predict the miR-103a-2-5p binding sites in the 3ʹ-UTR of LILRB3 mRNA. To construct the reporter vector LILRB3-MUT, mutations in the miR-103a-2-5p binding sites of the 3’-UTR region were cloned into pmirGLO Dual-luciferase vectors (IGEbio, China). The plasmid sequences and miRNA sequences are shown in Additional file 3: Table S3. 293 T cells were seeded into a 24-well plate. After incubated overnight, 293 T cells were co-transfected with the corresponding vectors and miRNA mimics respectively. Luciferase assays were performed 48 h after transfection using the Dual-Luciferase Reporter Assay System (Promega) according to the manufacturer’s instructions. For each transfected well, firefly luciferase activity was normalized to Renilla luciferase activity.

### Quantitative reverse transcription polymerase chain reaction (qRT-PCR)

Total RNA was extracted using TRIzol reagents (Invitrogen). About 1 ug of total RNA was reversely transcribed into cDNA with a PrimeScript RT reagent kit (Vazyme, Nanjing, China). qRT-PCR was tested by using a SYBR Green Dye detection system (Applied Biosystems). By using miRNA Stem-Loop RT primers, total RNA was reversely transcribed into cDNAs with the miRNA First-Strand cDNA Synthesis Kit (by stem-loop, Vazyme, Nanjing, China) and the miRNA U universal SYBR qPCR Master Mix (Vazyme, Nanjing, China) was used for miRNA qRT-PCR. GAPDH and U6 were examined as the internal control and reference genes for detecting mRNA and miRNA. The relative fold changes were analyzed by the 2^−ΔΔCt^ method. All the mRNA and miRNA primers were obtained from IGEbio (Guangzhou, China), and the sequences used are provided in Additional file 4: Table S4.

### Cell counting kit-8 (CCK-8) assay

CCK-8 assay was used to monitor cell growth. In brief, AML cells under different transfection (8 × 10^3^ cells/well) were seeded into 96-well plates for 0, 24, 48, or 72 h in 100 µl medium containing 10% FBS. Then, 10 µl CCK-8 solution (Dojindo, Kumamoto, Japan) was added to each well, followed by 4 h of incubation. Cell viability was detected at a wavelength of 450 nm. All experiments were performed in triplicate.

### Apoptosis assay

Annexin V-FITC/PI Apoptosis Detection Kit (Elabscience Biotechnology Co., Ltd.) was used to examine the apoptosis-mediated cell death. In brief, 1 × 10^6^ cells/well were seeded in 6-well plates. Next, the cells were collected and stained with Annexin V and propidium iodide (PI) solutions after transfecting miR-103-a-2-5p mimics or negative control (miR-NC) for 48 h. The apoptotic cells were detected by a Cytoflex Flow Cytometer (Beckman, USA) and quantified by Flowjo software following the manufacturer’s instructions.

### Cell cycle assay

AML cells were seeded in 6-well plates at 1 × 10^6^ cells/well, transfected with miR-103-a-2-5p mimics or negative control (miR-NC) for 48 h. Cells were collected, washed with PBS, and then fixed with precooled 75% ethanol at 4 ℃ overnight. Next, ethanol was extracted using centrifugation and washed with PBS thrice. Cells were incubated with 0.5 ml PI/RNase Staining Buffer (BD biosciences, USA) at room temperature for 30 min. Data were obtained by FACS analysis within 1 h. ModFit LT5.0 (Verity Software House, Topsham, ME) was utilized to ascertain the distribution of cells in different cell cycle phases.

### Colony formation assay

Transfected cells were counted and seeded at 500 cells/well in a 24-well plate. Methylcellulose (Sigma-Aldrich, USA) was dissolved in a culture medium at a concentration of 1%. After 14 days, cell colonies were imaged and counted.

### Transwell assay

Transwell assays were performed by using transwell chamber inserts (8 μm pore size; Corning Inc., Corning, NY). For cell migration, the upper chamber was supplemented with cells in the serum-free medium while the complete medium was added to the bottom chamber. After 24 h of incubation, migrated cells in the bottom chamber were imaged under a microscope and then all cells were collected for centrifugation. Next, cells were resuspended with the complete medium, and 10 µl CCK-8 solution was added to each well subsequently, followed by 4 h of incubation. Cell viability was detected at a wavelength of 450 nm.

### Western blot analysis

All prepared cells were homogenized in protein lysate buffer for 30 min at 4 ℃, and debris was removed by centrifugation at 12,000 *g* for 15 min at 4 ℃. The protein concentration was ascertained by a BCA protein assay kit (KeyGEN BioTECH, Jiangsu, China). Following the loading buffer addition, 30 µg of each protein sample was electrophoresed, transferred to PVDF membranes (0.2 μm; Millipore, Bedford, MA), and blocked for 2 h in silk milk. Subsequently, the anti-human primary antibody was applied to the membranes for immunoblotting overnight at 4 ℃. Following three times TBST washing, the membranes were incubated for 1 h at room temperature with horseradish peroxidase (HRP)-conjugated secondary antibodies. The HRP signal was then detected by an enhanced chemiluminescence kit (Fdbio Science, Zhejiang, China). Antibodies for GAPDH (10494-1-AP), Tubulin (66031-1-Ig), LILRB3 (18260-1-AP), BAX (50599-2-Ig), BCL2 (12789-1-AP), Caspase 3/p17/p19 (19677-1-AP), P21 (10355-1-AP), Cyclin D1 (60186-1-Ig), and CDK4 (11026-1-AP) were obtained from Proteintech (Wuhan, China). Antibodies for NRF2 (R1312-8), HO-1 (ET1604-45), SOD2 (ET1701-54), and NQO1 (JF440-1) were provided by HUABIO (Hangzhou, China).

### RNA sequencing

Total RNA from THP-1/miRNA NC and THP-1/miR-103a-2-5p mimics was extracted with TRIzol reagent, and the Illumina NovaSeq platform was used for sequencing. Using the DESeq2-EBSeq package, differentially expressed genes were identified by RefSeq ID.

### Flow cytometry analysis

AML cell lines and B-ALL cell lines were collected and suspended in PBS and then stained with PE anti-human LILRB3 antibody (PE-CD85a, 337703) for 30 min at 4 ℃ in the dark. AML cell lines were co-cultured in the U-bottom 96-well plate at different rations with activated PBMCs, which were purified by Ficoll density centrifugation from HLA-matched healthy donors. After 24 h or 72 h, for the detection of T cell apoptosis, cells were stained with CD3 (APC-CD3, 317317), CD8a (FITC-CD8a, 344703), Annexin V/PI and detected by FACS. For the detection of co-inhibitory receptors, cells were stained with CD3 (APC-CD3, 317317), CD8a (FITC-CD8a, 344703), Tim3 (PE-CD366,364805), CTLA4 (PE-CD152, 369603), LAG3 (PE-CD223, 369305), PD-1 (APC-CD279, 379207) antibodies for 0.5 h and detected by FACS. Flow human antibodies were provided by Biolengend.

### Reactive oxygen species (ROS) detection

2ʹ,7ʹ-Dichlorodihydro-fluorescein diacetate (DCFH-DA) (Elabscience Biotechnology Co., Ltd.) was used to measure intracellular reactive oxygen species (ROS) levels. Before the detection, DCFH-DA was dissolved in DMSO to make a stock solution. Then transfected cells were extracted from the growth media by centrifugation and exposed to a pre-warmed PBS containing DCFH-DA with a final concentration of 10 μM for 30 min at 37 ℃ in the dark. Next, the cells were washed twice using PBS and resuspended in the PBS. Lastly, flow cytometry measurements in the FITC channel were performed three times for each treatment. The data was evaluated with FlowJo software (FlowJo, USA).

### Glutathione (GSH) detection

AML cells were transfected with miR-103a-2-5p mimics or miR-NC mimics spreading in 12-well culture plates and cultured for 48 h. Then, the total GSH in the cells was detected by a Reduced Glutathione (GSH) Colorimetric Assay Kit according to the manufacturer’s instructions (Elabscience Biotechnology Co., Ltd.).

### Preparation and characterization of cationic liposomes (CLPs)

To efficiently and steadily deliver miR-103a-2-5p into mouse tumor tissue, CLPs-miR-103a-2-5p were designed. CLPs with molar proportions of 1,2-dioleoyl-3-trimethylammonium propane (DOTAP) (Macklin, China), Distearoyl Phosphatidylcholine (DSPC) (Macklin, China), Cholesterol (Macklin, China), DSPE-PEG2000 (WEIHUA BIO, Guangzhou), and DSPE-PEG-MAL5000 (WEIHUA BIO, Guangzhou) were produced based on the thin-film hydration method. Eventually, the solution was extruded 3 times through polycarbonate membranes with pore sizes of 0.45 µm and 0.22 µm to obtain colloidally stable liposomes. These liposomes were then complexed with Fam-labeled or label-free miR-103a-2-5p to form colloidally stable CLPs-miR-103a-2-5p by a simple self-assembly method. The zeta potential, hydrodynamic size, and dynamic light scattering of CLPs and CLPs-miRNA were measured by Nanoparticle Size and Zeta Potential Analyzer (Litesizer 500, Anton Paar, Austria), and the surface morphologies were observed by transmission electron microscopy (TEM, JEM-2100, Japan). Fam-labeled CLPs-miRNA were visualized by confocal microscope (Nikon, AX NIS-Elements 5.4, Shanghai, China).

### Xenograft assay

To assess the inhibitory effect of CLPs-miR-103a-2-5p on AML, the mouse subcutaneous tumor model was established. The female BALB/c-nu mice, aged 6 weeks, were acquired from BIOSYTOGEN (Jiangsu, China). Briefly, approximately 6 × 10^6^ THP-1 cells were resuspended in 100 µl of PBS and then subcutaneously injected into the right back of nude mice. The tumor volumes of the mice were measured (the formula for calculating the tumor volume was V = a × b^2^/2, where a represents the length and b is the width). While the volume of subcutaneous tumor approached approximately 100 mm^3^, PBS, CLPs-miR-NC, and CLPs-miR-103a-2-5p (miRNA, 5 µg per mouse) were injected twice every 3 days for 1 month into mice (n = 4) via the peritumoral, and the body weights and tumor volumes of the mice were measured before each injection. Before the mice were sacrificed, whole blood was collected to analyze liver and kidney function-related indicators and routine physical examination via the biochemical assay kits. To form tumors in the bone marrow as in AML patients, we established the tail vein xenograft model. The NOD.CB17-Prkdcscid/l2rgtm1/Bcgen (B-NDG)mice (6 weeks, female) were purchased from BIOSYTOGEN (Jiangsu, China) and maintained under specific pathogen-free (SPF) conditions. 1 × 10^6^ GFP-tagged THP-1 cells were injected into B-NDG mice via tail vein (day 0), and 7 days later, CLPs-miR-103a-2-5p or CLPs-miR-NC (at 10 µg/20 *g* body weight, every 2 days for 2 weeks) or PBS was injected into the tail vein. The mice were sacrificed 35 days later to remove the spleen and bone marrow for FACS analysis of tumor burden, and in addition, the survival time of the mice was recorded. All animal studies were approved by the Ethical Committee for Animal Research of Zhujiang Hospital of Southern Medical University.

### Hematoxylin and eosin (H&E) staining

The primary organs (liver, spleen, kidneys, lungs, and heart) were harvested for H&E staining. The sections were completely submerged in xylene for 30 min and then rehydrated with different concentrations of ethanol for 5 min of each. Submerged and washed with PBS 3 times, 3 min each time. Immersed in hematoxylin solution and stained for 5 min. Soaked in 1% hydrochloric acid alcohol for 1 s. After being washed with water, the sections were stained for 3 min with eosin solution, dehydrated with graded alcohol, and transparented in xylene. Lastly, a neutral resin was used to mount the sections. Representative images were taken after section dyeing using a Leica light microscope.

### Immunohistochemical (IHC) analysis

Subcutaneous tumors were collected for IHC staining assay. Tumor tissue was fixed, and dehydrated for paraffin-embedded, and then 5 µm sections were cut from each paraffin block using a microtome. The sections were then dewaxed with xylene and rehydrated in alcohol. 3% H_2_O_2_ was used to block endogenous peroxidase and microwave heating was used to retrieve the antigen. Nonspecific antigen was blocked for 30 min at 37 ℃ using 5% Bovine Serum Albumin (BSA) and then the sections were incubated with a specific primary antibody at 4 ℃ for an entire night. For the remaining steps, the sections were incubated with corresponding secondary antibodies for 1 h at 37 ℃, and then stained with DAB Color Development Kit (ZSGB-BIO, ZLI-9017). Secondary antibodies for HRP AffiniPure Goat Anti-Rabbit IgG and HRP AffiniPure Goat Anti-Mouse IgG were provided by Fdbio (Hangzhou, China). Representative images were taken after section dyeing using a Leica light microscope. The resulting area and cell measurement were quantified using ImageJ Toolbox software analysis.

### Statistical analysis

Experimental data are shown as the mean ± standard deviation (mean ± S.D.) of three independent repeated experiments and analyzed by the SPSS (version 17.0) software program. Two group comparison data were analyzed by the independent samples t test. Multiple group comparison data were studied by one-way ANOVA and Tukey’s post-hoc test. The Kaplan–Meier method was used to analyze survival curves and survival rates were compared by the log-rank test. Statistical significance was determined as *P < 0.05, **P < 0.01, ***P < 0.001, **** P < 0.0001, or no significant difference (ns).

## Results

### Increased LILRB3 expression is observed in AML patients and cell lines

**Fig. 1:**
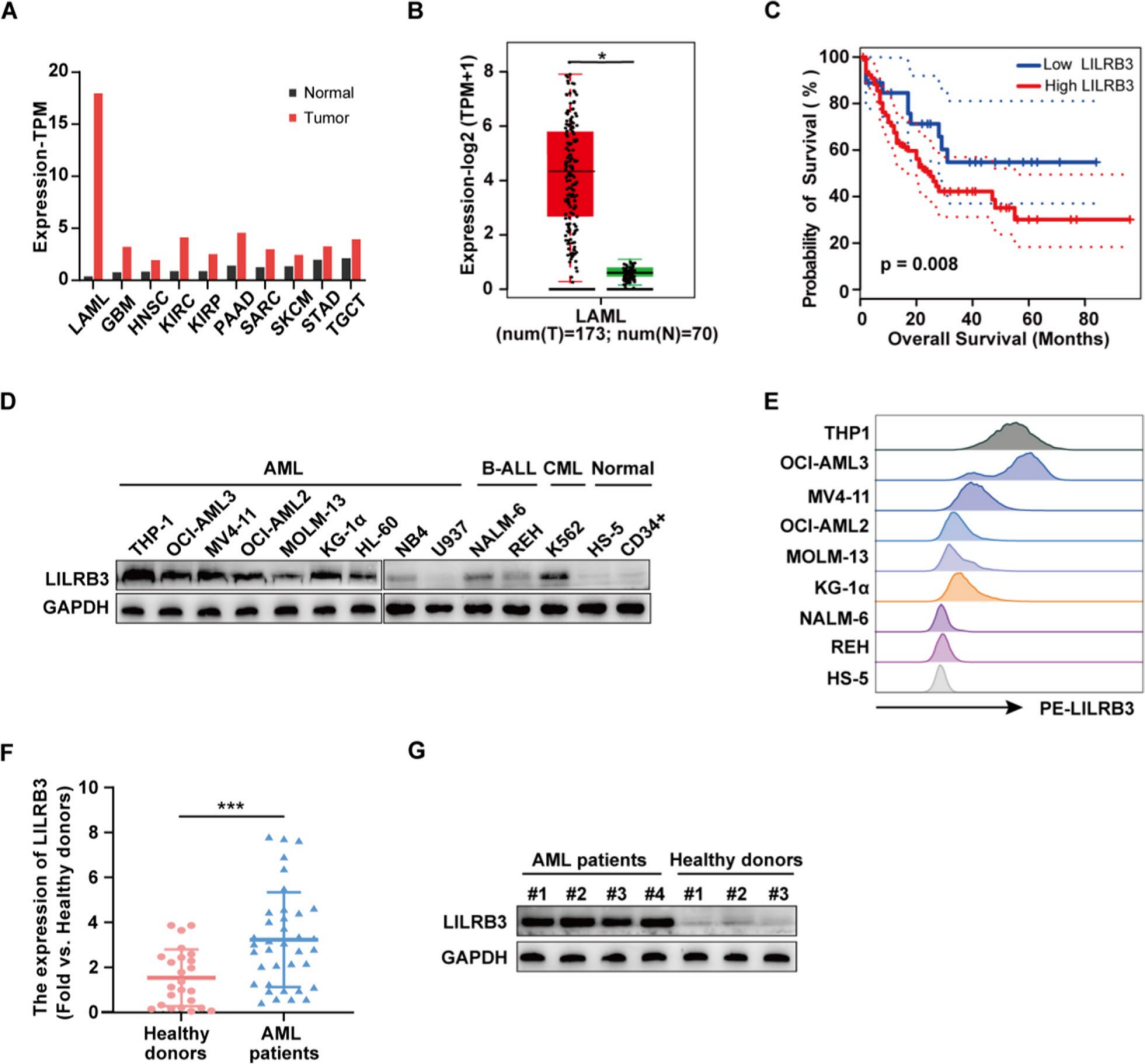
LILRB3 is highly expressed in patients with AML and AML cells. **A** The expression profile of LILRB3 across TCGA datasets. Images were obtained from the GEPIA online database (http://gepia2.cancer-pku.cn/). *LAML* acute myeloid leukemia, *GBM*:glioblastoma multiforme, *HNSC* head and neck squamous cell carcinoma, *KIRC* kidney renal clear cell carcinoma, *KIRP* kidney renal papillary cell carcinoma, *PAAD* pancreatic adenocarcinoma, *SARC* sarcoma, *SKCM* skin cutaneous Melanoma, *STAD* stomach adenocarcinoma, *TGCT* testicular germ cell tumors. **B** Expression levels of LILRB3 in AML from TCGA datasets. Tumor: acute myeloid leukemia; normal: normal donors. **C** Kaplan–Meier overall survival curves for 173 AML patients classified according to relative LILRB3 expression level. Patients with higher LILRB3 had a shorter survival. **D** The LILRB3 protein level in a panel of malignant hematological cells, normal bone marrow cell lines (HS-5), and peripheral blood CD34 + cells from healthy donors. **E** LILRB3 expression on the AML cell surface was higher than that on B-ALL cell lines and HS-5. **F**–**G** LILRB3 mRNA and protein levels in BMMCs of AML patients and healthy donors in our clinical samples. *P < 0.05, **P < 0.01, ***P < 0.001

LILRB3 expression in AML was first examined and verified. LILRB3 was up-regulated in a variety of cancers, including AML (Fig. 1A) [7, 19, 20], and was significantly increased in AML patients compared to normal people, based on GEPIA data (Fig. 1B). Furthermore, the GEPIA results showed that AML patients with lower levels of LILRB3 expression have longer survival times (Fig. 1C).

To verify the bio-informatics results, we examined the expression of LILRB3 protein in AML, B-ALL, and CML cell lines, HS-5, and CD34 + cells of healthy human peripheral blood. As expected, the expression of LILRB3 was significantly higher in AML cells (i.e., THP-1, OCI-AML2, OCI-AML3, and MV4-11) than in HS-5 cells and CD34 + cells. Interestingly, we found the protein level of LILRB3 was also higher in K562 cells than in the control cells, which is consistent with the reported expression of LILRB3 in certain myeloid cells (Fig. 1D) [20]. In addition, the expression of LILRB3 on the AML cell surface was higher than that on B-ALL cell lines and HS-5 (Fig. 1E). The representative AML cell lines, THP-1, OCI-AML3, OCI-AML2 and MV4-11, were chosen to assess the functional roles of miRNAs in the subsequent experiment. To evaluate the clinical relevance of LILRB3, BMMCs from AML patients and healthy donors were collected. We identified LILRB3 expression using qRT-PCR assay. Consistent with the above data, the expression of LILRB3 was significantly up-regulated in AML patients than in healthy donors (Fig. 1F). Moreover, western blot analysis indicated that LILRB3 protein was upregulated in AML samples (Fig. 1G). These findings suggested that poor survival was associated with the upregulation of LILRB3 in AML.

### Evaluation and verification of candidate target miRNAs for LILRB3

Numerous studies have demonstrated the significant role miRNAs play in AML [10, 21, 22]. MiRNAs typically work by interacting with downstream target mRNA. To determine whether LILRB3 modulates the tumor growth and cell proliferation of AML by interacting with miRNAs, we looked up bioinformatics websites to predict the target miRNAs of LILRB3.

**Fig. 2:**
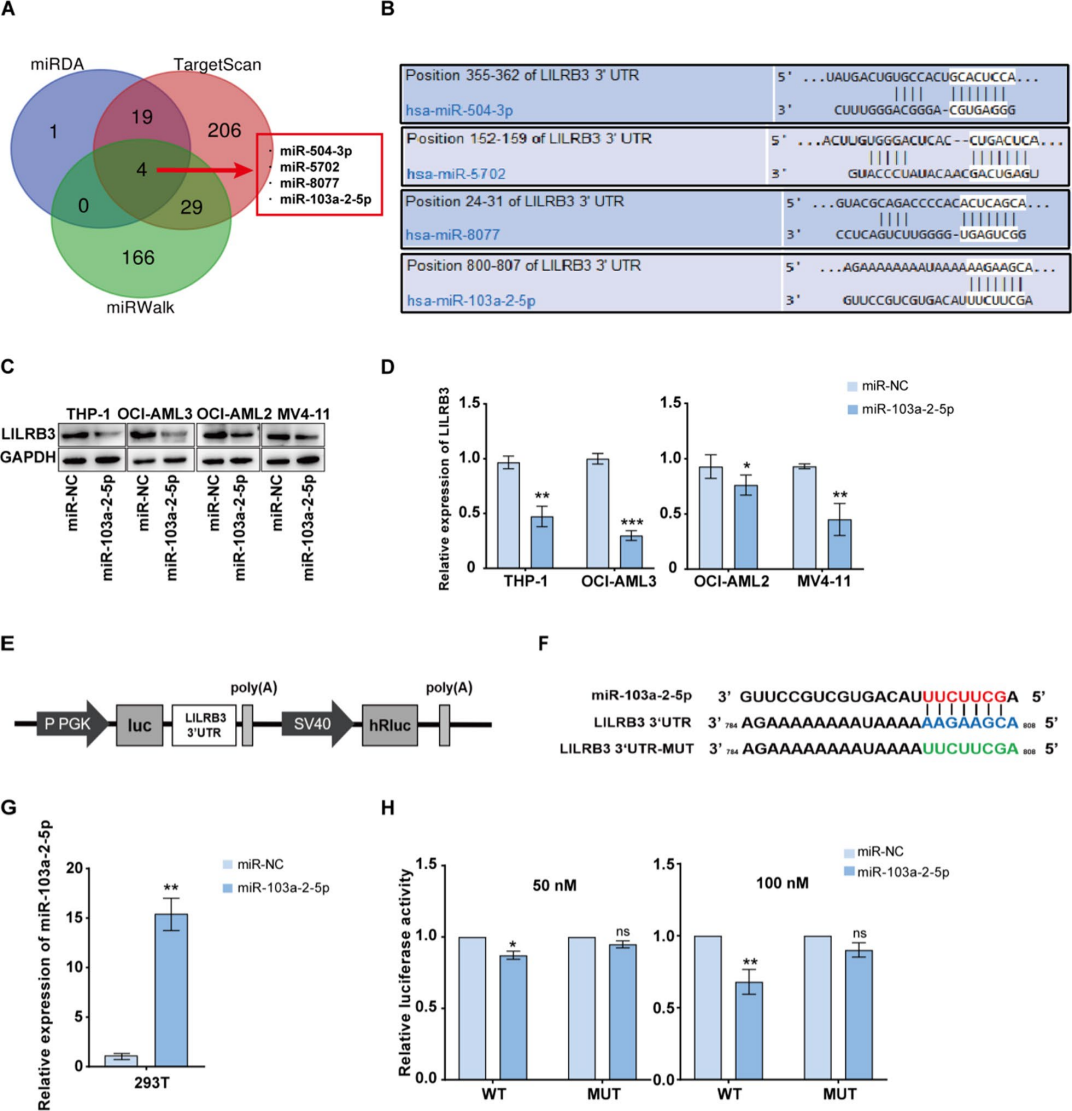
Screening and verification of candidate LILRB3 upstream target miRNAs, and miR-103a-2-5p was selected. **A** Venn diagram of the target miRNAs of LILRB3 based on miRDB, TargetScan, miRWalk. **B** A potential miR-504a-3p, miR-5702, miR-8077, and miR-103a-2-5p binding site in the 3ʹ-UTR of LILRB3 was predicted using TargetScan (https://www.targetscan.org/). The expression of LILRB3 at the protein (**C**) and mRNA (**D**) levels was measured by qRT-PCR and western blotting analysis. (**E**, **F**) The sequence of luciferase reporter vector. Luciferase reporter gene assays were performed to confirm the direct targeting of LILRB3 to miR-103a-2-5p. **G** The qRT-PCR assay examined the expression of miR-103a-2-5p in HEK293T cells transfected with control miRNA (miR-NC) or miR-103a-2-5p. **H** Cells were transfected with either luciferase reporter vector containing the LILRB3-3ʹUTR wild-type or mutant in the target sites in the presence or absence of precursors of miR for 48 h followed by measuring the luciferase activity. All results are presented as the mean ± SD, *P < 0.05, **P < 0.01. Cell experiments were performed three times independently

Three distinct algorithms (TargetScan, miRDB, miRWalk) were principally used to identify candidate miRNAs involved in regulating LILRB3. Four miRNAs with target sites present in all three algorithms were chosen as candidate miRNAs (Fig. 2A, B). To further confirm the expression of the four miRNAs, we discovered that miR-103a-2-5p expression level in AML cell lines was lower than that in HS-5 using qRT-PCR (Additional file 6: Fig. S1A). Further, AML cells were transfected with negative control mimics and four miRNA mimics respectively, and then we detected the level of LILRB3. According to qRT-PCR and western blot results, the miR-103a-2-5p group’s LILRB3 mRNA and protein levels were significantly lower than those of the miR-NC group (Fig. 2C, D), while other miRNAs were not (Additional file 6: Fig. S1B-E).

To further verify that miR-103a-2-5p binds directly to LILRB3 mRNA, we performed a dual-luciferase reporter assay by constructing the WT or MUT LILRB3 3ʹ-UTR reporter vector (Fig. 2E, F). The indicated vectors and miRNA mimics were co-transfected into 293 T cells respectively. MiR-103a-2-5p expression in the miR-103a-3-5p mimics-transfected group is higher than that in the NC mimic group in 293 T cells, which indicated that the transfection was successful (Fig. 2G). In 293 T cells, the luciferase activities of WT LILRB3 reporter were decreased by overexpression of miR-103a-2-5p mimic, but not those of the MUT LILRB3 reporter (Fig. 2H). We also detected the level of miR-103a-2-5p in K562 by qRT-PCR, while it was higher than that in HS-5 (Additional file 6: Fig. S1H). All of these results indicated that miR-103a-2-5p directly targeted LILRB3 3ʹ-UTR, which could have an effect on AML.

### MiR-103a-2-5p expression is down-regulated in AML patients

**Fig. 3:**
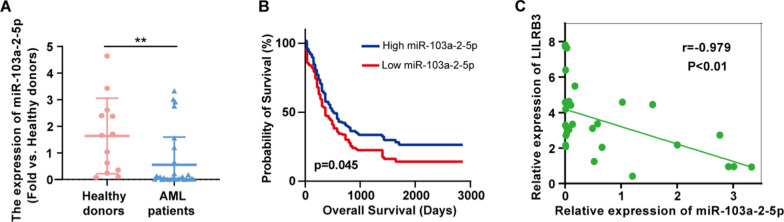
The expression of miR-103a-2-5p in AML. **A** miR-103a-2-5p expression in BMMCs of AML patients and healthy donors in our clinical samples. **B** Kaplan–Meier overall survival curves for 381 patients with AML classified according to relative miR-103a-2-5p expression level. Patients with lower miR-103a-2-5p had shorter survival. **C** The correlation between miR-103a-2-5p expression and LILRB3 expression was analyzed using Spearman’s rank correlation analysis. All results are presented as the mean ± SD, *P < 0.05, **P < 0.01. Cell experiments were performed three times independently

To assess the clinical significance of miR-103a-2-5p, BMMCs from AML patients and healthy donors were collected. qRT-PCR analysis showed significantly lower levels of miR-103a-2-5p in AML patients than in healthy donors (Fig. 3A). According to the data from TCGA, 381 cases were divided into two groups based on the expression of miR-103a-2-5p: a low miR-103a-2-5p group (miR-103a-2-5p expression below the median value, n = 194) and a high miR-103a-2-5p group (miR-103a-2-5p expression above the median value, n = 187). AML patients with higher expression of miR-103a-2-5p had better survival times (Fig. 3B). Further, we examined the correlation between the expression of LILRB3 and miR-103a-2-5p. As shown in Fig. 3C, higher LILRB3 mRNA level was associated with lower miR-103a-2-5p level, suggesting an inverse relationship between the two in AML patients.

### Overexpression of miR-103a-2-5p inhibits AML cell proliferation and cell cycle and promotes cell apoptosis in vitro

**Fig. 4:**
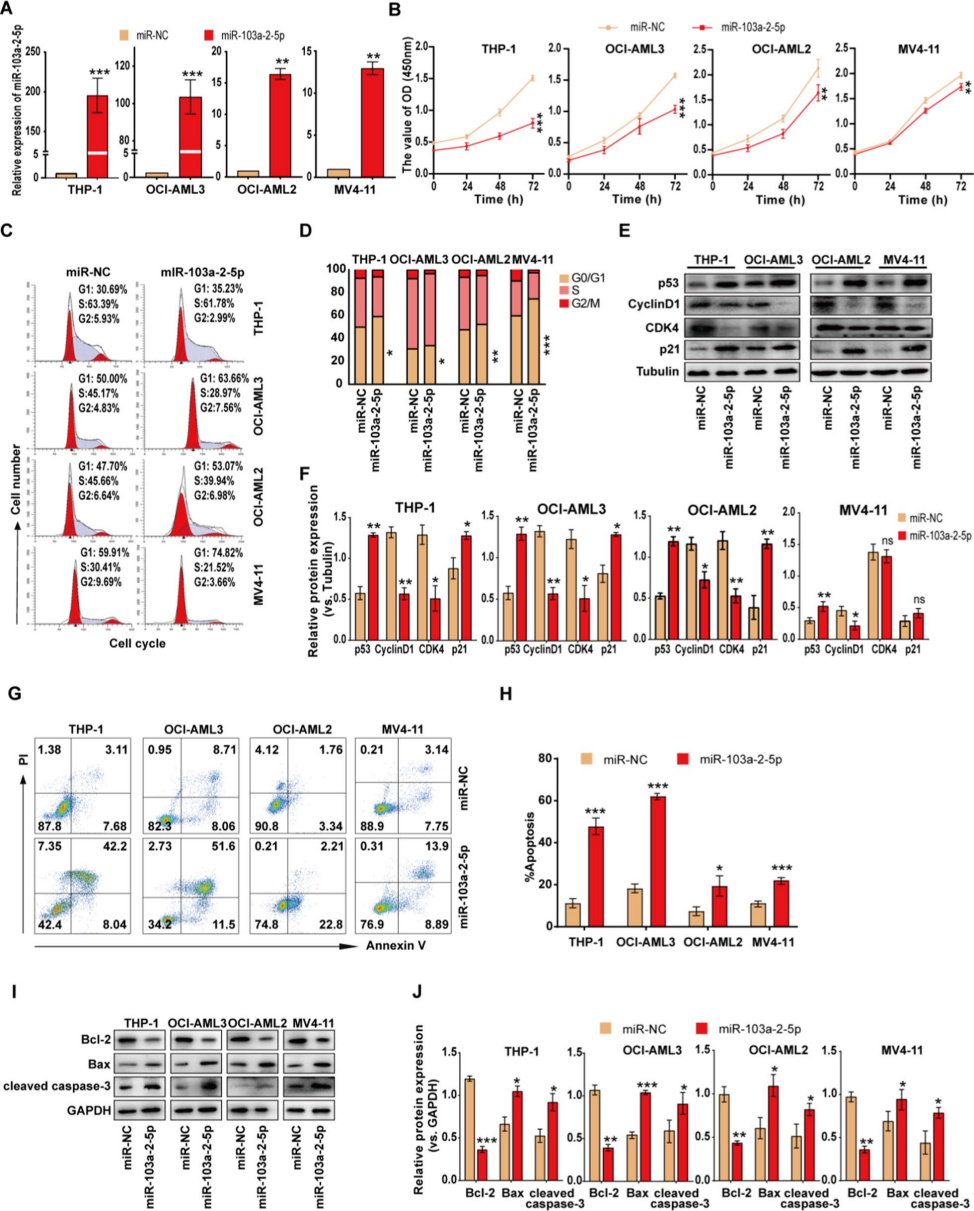
Overexpression of miR-103a-2-5p inhibits AML cell proliferation and cell cycle and promotes cell apoptosis in vitro. **A** After transfection with miR-103a-2-5p mimics or control miRNA (miR-NC) in THP-1, OCI-AML3, OCI-AML2, and MV4-11 cells for 48 h, the qRT-PCR assay was conducted to evaluate transfection efficiency. **B** After transfection with miR-103a-2-5p mimics or control miRNA (miR-NC) for 48 h, the CCK-8 assay was used to test cell proliferation. **C**, **D** Cell cycle distribution was measured by flow cytometry. **E**, **F** Levels of the cycle-related proteins, p21, cyclinD1, CDK4, and p53 were detected by western blot analysis. **G**, **H** Cell apoptosis was detected by flow cytometry. **I**, **J** Levels of the apoptosis-related proteins, cleaved-caspase3, Bax, and Bcl-2 were detected by western blot analysis. All results are presented as the mean ± SD, *P < 0.05, **P < 0.01, ***P < 0.001 vs. control miRNA (miR-NC). Cell experiments were performed three times independently

LILRB3 has been shown to promote the development of AML and regulate T-cell antitumor immunological responses via the TRAF2–cFLIP–NF-κB signaling pathway [7]. Considering that we have demonstrated that miR-103a-2-5p mimics could reduce LILRB3 mRNA and protein levels and miR-103a-2-5p is downregulated in AML patients and cell lines, it is necessary to evaluate whether enhanced miR-103a-2-5p expression would affect the biological activity of AML cells by reducing the expression of LILRB3. To achieve this, AML cells were transfected with negative control mimics (“miR-NC group”) and miR-103a-2-5p mimics (“miR-103a-2-5p group”) respectively. Firstly, we performed qRT-PCR to examine miR-103a-2-5p expression in AML cells after transfection. MiR-103a-2-5p was highly expressed in the miR-103a-2-5p group compared to the miR-NC group (Fig. 4A). Subsequently, CCK-8 assays demonstrated that proliferation rates of AML cells were inhibited after transfection with miR-103a-2-5p mimics (Fig. 4B).

In addition, AML cells transfected with miR-103a-2-5p mimics were further examined by flow cytometry to assess their cell cycle stage and apoptosis. As shown in Fig. 4C, D, overexpression of miR-103a-2-5p in AML cells induced G0/G1 cell cycle arrest. CDK 4 and cyclinD1, the major mediators of the cellular transition to the S phase, are essential for the initiation, development, and survival of multiple cancers, including AML [23, 24]. P21 and p53 are also cell cycle regulators involved in the regulation of cell cycle arrest. Using the western blot method, we discovered that following miR-103a-2-5p mimics transfection, the expression of CDK4 and cyclinD1 decreased while that of p21 and p53 protein increased (Fig. 4E, F).

Data from the apoptosis analysis also demonstrated that transfection of miR-103a-2-5p mimics resulted in a significant increase in the apoptosis rate in AML cells (Fig. 4G, H). According to western blot analysis, the expression of the anti-apoptotic protein Bcl2 in the cells transfected with miR-103a-2-5p mimics was downregulated while that of Bax and cleaved-caspase-3 protein was upregulated (Fig. 4I, J).

### Upregulation of miR-103a-2-5p attenuates clonality and the invasive abilities of AML cells

Furthermore, transwell migration assays showed that the number of migrated THP-1 or MV4-11 cells was significantly reduced (Additional file 7: Fig. S2A) after transfection with miR-103a-2-5p mimics. Similarly, the colony formation assay showed a significant decrease in the clonogenic capacity of THP 1 and MV4-11 cells (Additional file 7: Fig. S2B).

### Overexpression of miR-103a-2-5p enhances CD8 + T cell survival and reduces inhibitory receptor generation

**Fig. 5:**
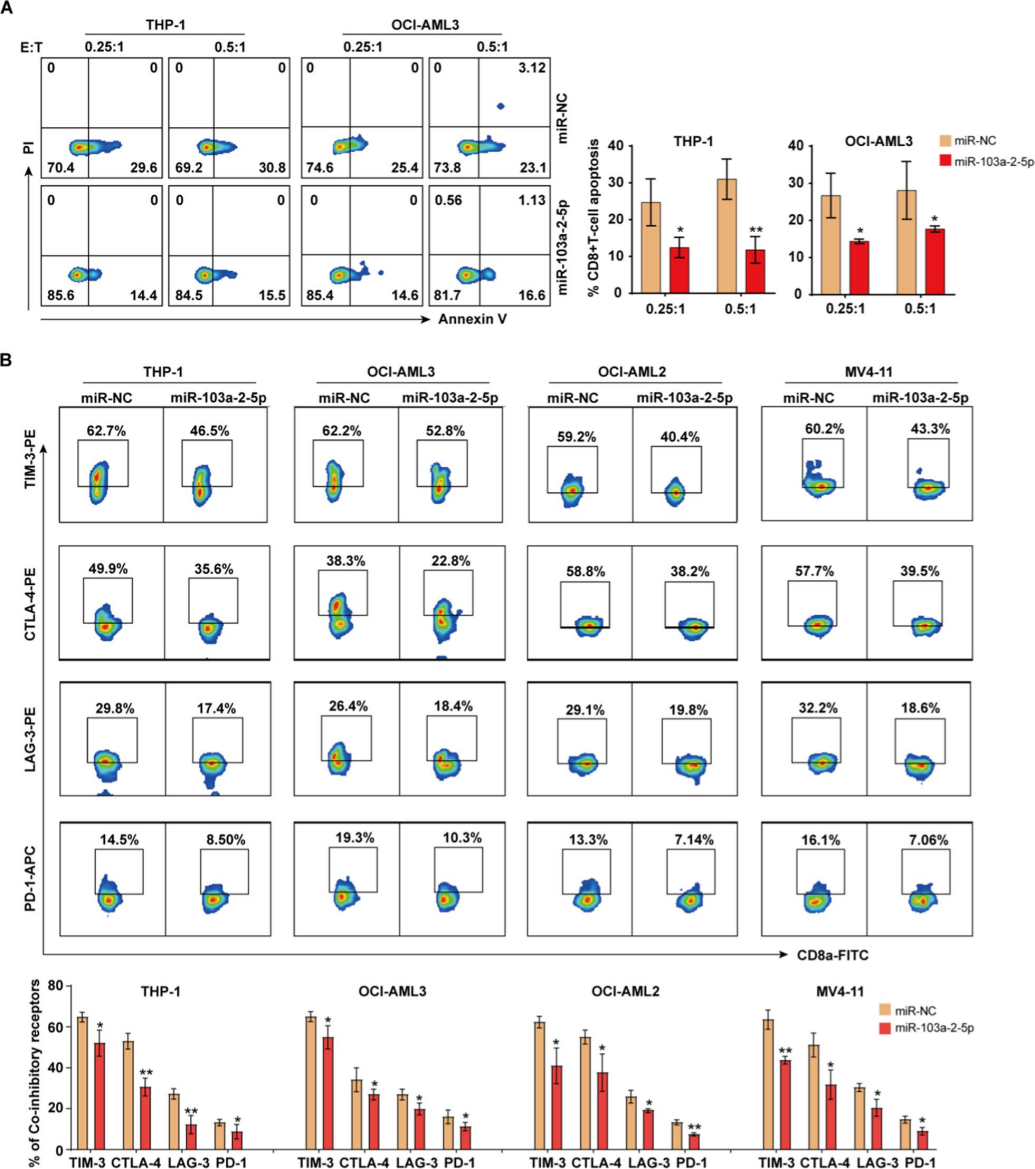
MiR-103a-2-5p enhances CD8 + T cells survival and reduces inhibitory receptor generation. Tumor cells and PBMCs were co-cultured in the U-bottom 96-well plate for 24 h, and (**A**) CD8 + T cell apoptosis was detected by flow cytometry. **B** The expression of immune checkpoints on T cells was detected by flow cytometry. All results are presented as the mean ± SD, *P < 0.05, **P < 0.01, ***P < 0.001 vs. control miRNA (miR-NC). Cell experiments were performed three times independently

As we know, CD8 + T lymphocytes circulate in the blood and perform cytotoxic functions. Many studies have shown that LILRB3 could cause immune escape as an immune checkpoint [7, 8, 25, 26]. Therefore, to mimic the in vivo situation and verify the impact of the miR-103a-2-5p on CD8 + T cells, we co-cultured AML cells transfected with miR-103a-2-5p mimics or miR-NC mimics and HLA-matched PBMCs (Additional file 5: Table S3) for 24 h or 72 h and then examined apoptotic CD8 + T cells by staining the sample with Annexin V-APC/PI-PE. After gating lymphocytes, single cells, and CD8 + T cells, we calculated the percentage of apoptotic CD8 + T cells. The miR-103a-2-5p group exhibited a considerably lower percentage of apoptotic CD8 + T cells than the miR-NC group according to the flow cytometry results (Fig. 5A; Additional file 8: Fig. S3A).

To further determine the potential mechanism of miR-103a-2-5p on CD8 + T cells, we also performed flow cytometry to analyze immune checkpoint receptors including PD-1, CTLA-4, LAG-3, and TIM-3 on T cells in the miR-103a-2-5p/NC mimics AML co-culture system. The flow cytometry results showed that the expression of immune checkpoints in the miR-103a-2-5p group was lower than that in the miR-NC group (Fig. 5B; Additional file 8: Fig. S3B).

### MiR-103a-2-5p suppresses LILRB3 function by suppressing LILRB3 protein level and affects apoptosis through the Nrf2/HO-1 pathway

**Fig. 6:**
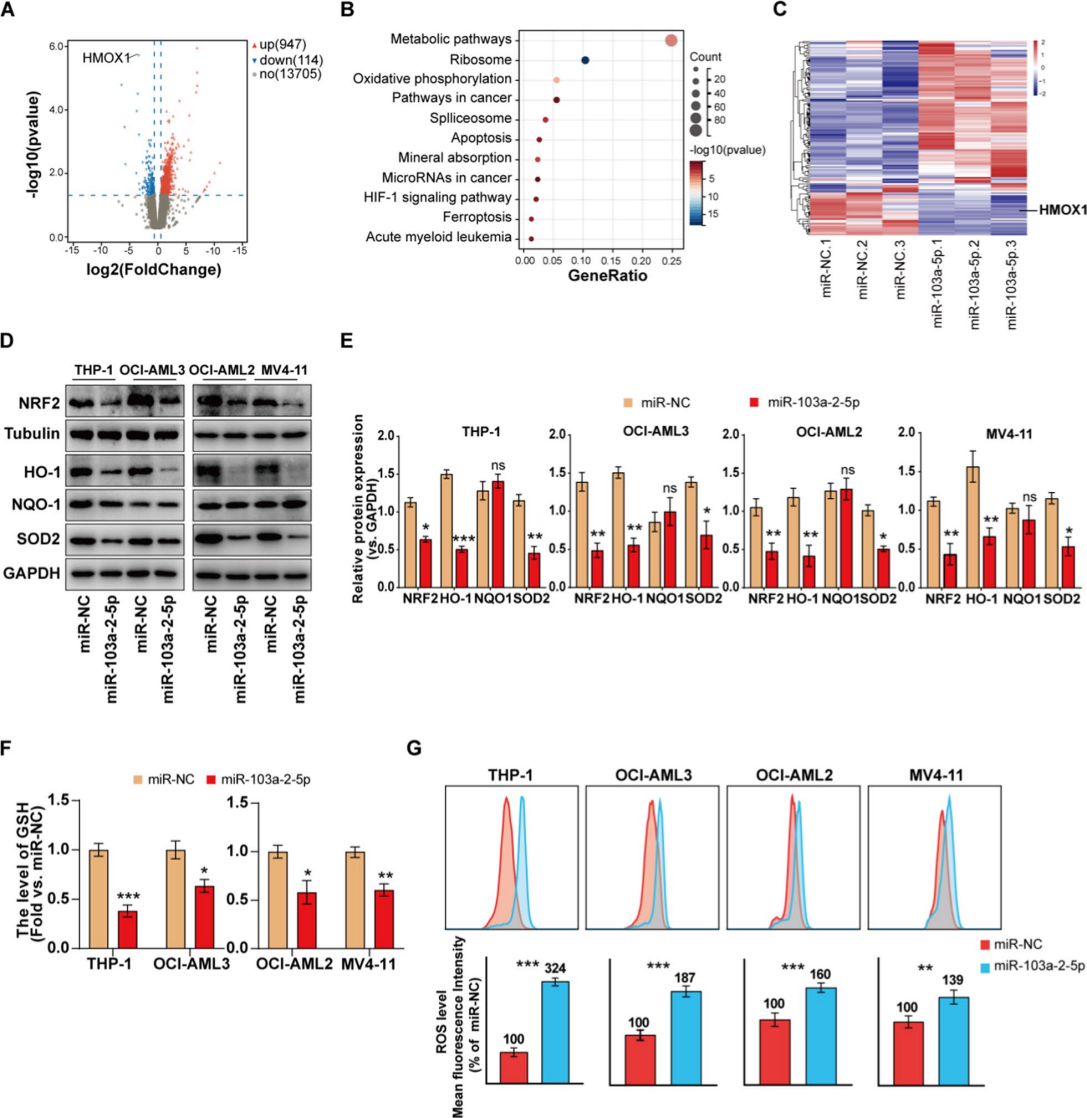
Mechanism of miR-103a-2-5p promotes AML cell apoptosis. THP-1 cells were transfected with control miRNA (miR-NC) or miR-103a-2-5p mimics for 48 h, **A** Volcano plot showing differential genes of THP-1 cells. **B** KEGG enrichment analysis of RNA-seq data from THP-1 cells. **C** Heatmap of the gene expression pattern of the Metabolic pathway in THP-1 cells. **D**, **E** Protein level associated with the Nrf2/HO-1 pathway was detected by western blot. **F** Total GSH in the cells was detected by a GSH assay kit according to the manufacturer’s instructions. **G** Flow cytometric histograms indicate DCF fluorescence in the AML cell lines. All results are presented as the mean ± SD, *P < 0.05, **P < 0.01, ***P < 0.001 vs. control miRNA (miR-NC). Cell experiments were performed three times independently

To delve deeper into the underlying mechanism by which miR-103a-2-5p promotes AML progression, comparative transcriptomic and functional analyses of miR-NC *vs.* miR-103a-2-5p THP-1 cells were performed. MiR-103a-2-5p overexpression led to the up-regulation of 947 genes and the down-regulation of 114 genes (P < 0.05, Fig. 6A). With miR-103a-2-5p mimics transfected, we discovered changed expression of more than 80 genes linked to the metabolic pathway among these transcripts (Fig. 6B). Heme oxygenase-1, or HMOX1 (HO-1), is a crucial enzyme in heme catabolism and is involved in the cellular response to oxidative stress, which is intimately associated with human metabolic progress [27]. As the volcano map and heatmap shown in Fig. 6A andC, the expression level of HO-1 was markedly suppressed in the miR-103a-2-5p group. The production of HO-1 is regulated by the NRF2 antioxidant response element signaling pathway [28]. It has been reported that NRF2 suppression could decrease HO-1 expression and inhibit viability in AML [29–31]. To further validate the changes of the Nrf2/HO-1 pathway after transfecting miR-103a-2-5p mimics, the expression levels of NRF2, HO-1, and a few downstream signaling molecules were measured by qRT-PCR and western blot. In vitro experiments, the protein level of NRF2, HO-1, and SOD2 was significantly decreased, while NQO1 did not change (Fig. 6D, E). Furthermore, our findings also showed that in comparison to control groups, overexpression of miR-103a-2-5p dramatically reduced the mRNA levels of NRF2, HO-1, and SOD2 (Additional file 9: Fig. S4).

### MiR-103a-2-5p consumes GSH and boosts ROS generation

GSH, an important intracellular antioxidant, can eliminate excessive ROS and maintain intracellular redox balance. However, excessive intracellular ROS might promote cell apoptosis and regulate tumor proliferation [32]. Consequently, consuming GSH and boosting ROS generation is a potential treatment strategy for AML. It is worth mentioning that miR-103a-2-5p decreased the GSH levels in four AML cells (Fig. 6F). The ROS detector DCFH was utilized to assess the production of ROS by evaluating the intensity of fluorescence. MiR-103a-2-5p, on the other hand, increased the level of ROS (Fig. 6G).

### CLPs are stable nanoparticles and deliver miRNAs into cells

**Fig. 7:**
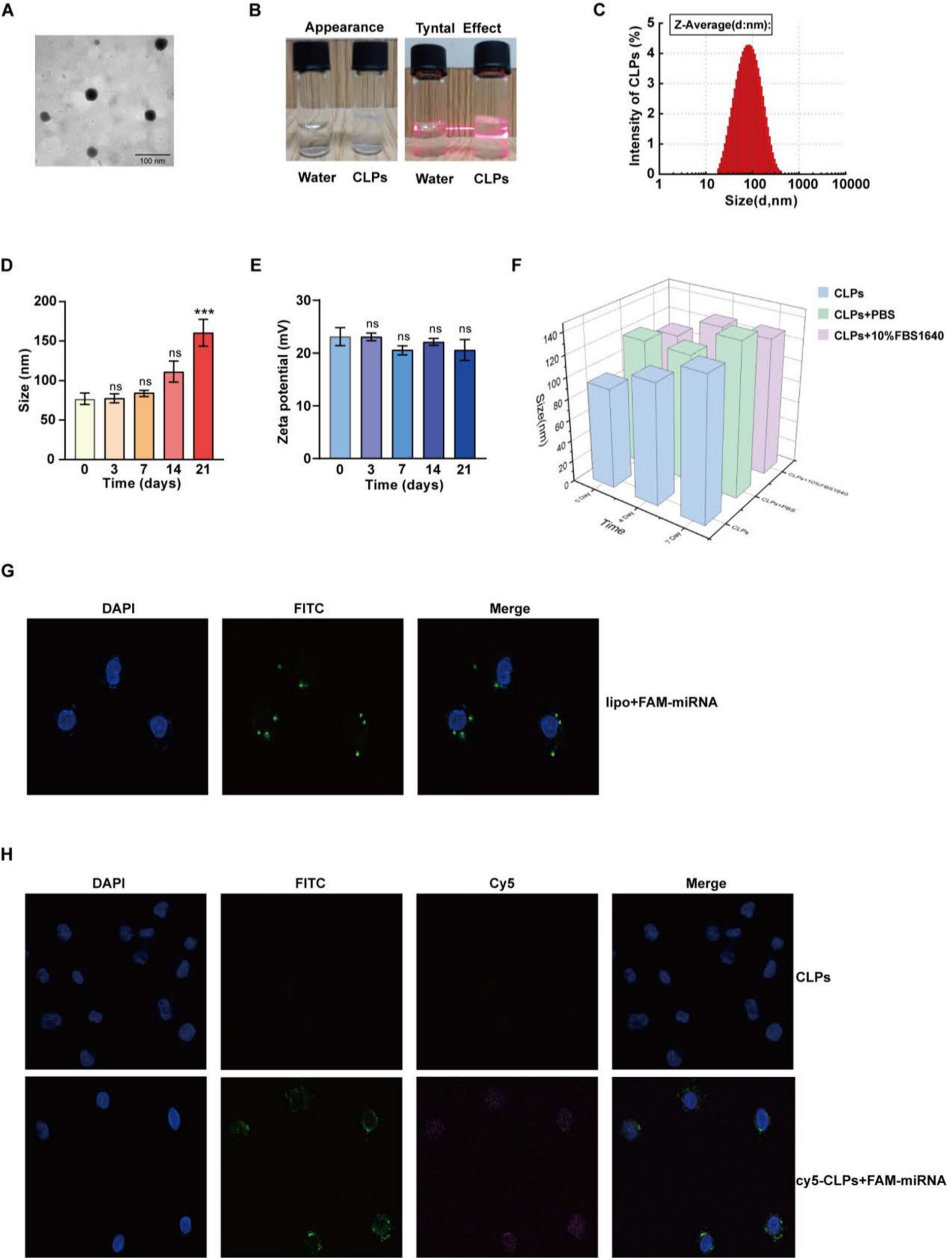
Characterization of Cationic liposomes (CLPs). **A** Representative TEM image of CLPs. Scale bar = 100 nm. **B** Appearance (left) and Tyndall effect (right) of CLPs, compared with water. **C** The particle size distributions histogram of CLPs obtained from dynamic light scattering (DLS). Stability of CLPs (**D**) particle size and (**E**) zeta potential. **F** Three-dimensional diagram of CLPs size in different kinds of solutions. **G**, **H** Representative immunofluorescent visions of CLPs [red], miR-103a-2-5p [green], and nucleus counterstained with DAPI [blue]

To improve delivery efficiency, we synthesized CLPs and created a liposome-packed miRNA system called CLPs-miR. The spherical shape of the CLPs was visible under a transmission electron microscope (negatively stained by phosphotungstic acid) (Fig. 7A). Compared with water, the colloidal solution of CLPs was transparent and had a clear Tyndall effect, indicating the presence of abundant nanoparticles that were completely dispersed in aqueous media (Fig. 7B). The particle size distributions of the CLPs were detected by the dynamic light scattering (DLS). As shown in Fig. 7C and Table 1, the characterization of CLPs was less than 100 nm. The hydrodynamic diameter of CLPs-miR was slightly larger than that of CLPs. The zeta potential of CLPs-miR decreased significantly, which indicated that miRNAs could diminish the positive charge of the CLPs. Although the particle size of CLPs increased slightly with time, the fluctuation did not exceed 20 nm with better stability (Fig. 7D). Similarly, the zeta potential of CLPs and three-dimensional diagram of CLPs size in different kinds of solutions were stable over time (Fig. 7E, F). To confirm that miRNAs could be delivered into cells by Lipofectamine 3000 Reagent, Fam-labeled miRNA was used in vitro experiments. The fluorescent dye DAPI penetrated the cell membrane and bound to DNA in the nucleus to emit blue fluorescence. Visualized by confocal microscope, the labeled nuclei were round or oval with a complete karyotype and Fam-miR transported into cells were evenly distributed around the nucleus of AML cells, verifying that miRNA could be delivered into cells (Fig. 7G). Next, to confirm that CLPs could deliver miRNAs into cells, Cy5.5-labeled CLPs were used to deliver FAM-miR. As shown in Fig. 7H, the cytoplasm of Cy5.5/Fam-labeled CLPs-miR group emitted a red fluorescen and Fam-miR were evenly distributed around the nucleus of AML cell, supporting that CLPs could be uptaken by cell membranes and transfer the miRNA into the cells.

**Table 1:** The characterization of the CLPs and CLPs-miR-103a-2-5p

Nano particle	Size (nm)	Zeta (mV)	PDI
CLPs	77.59 ± 0.94	23.11 ± 1.69	0.246
CLPs-miR	84.65 ± 1.72	13.64 ± 3.68	0.258

### CLPs-miR-103a-2-5p affects the proliferation of AML cells in vivo

**Fig. 8:**
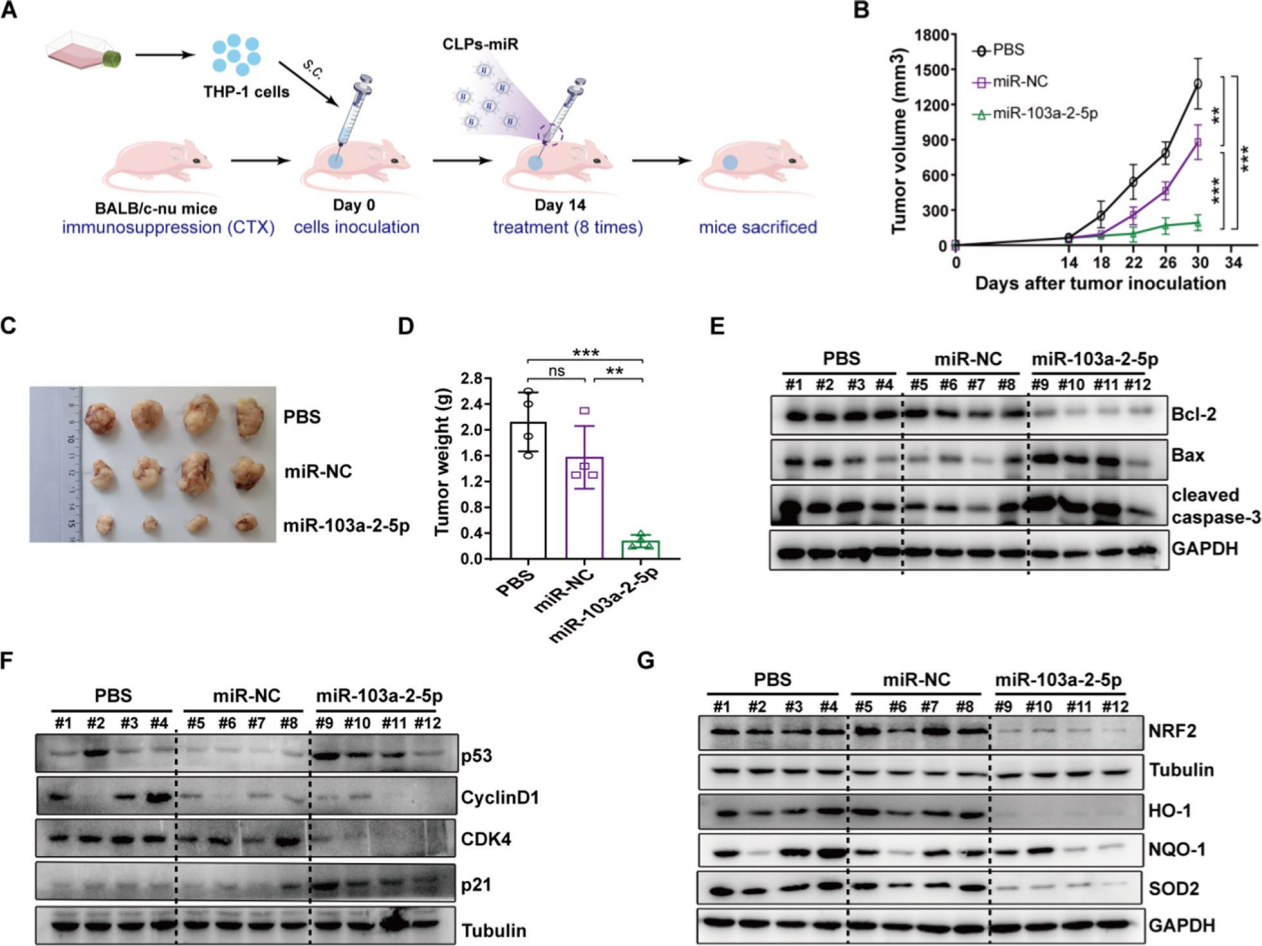

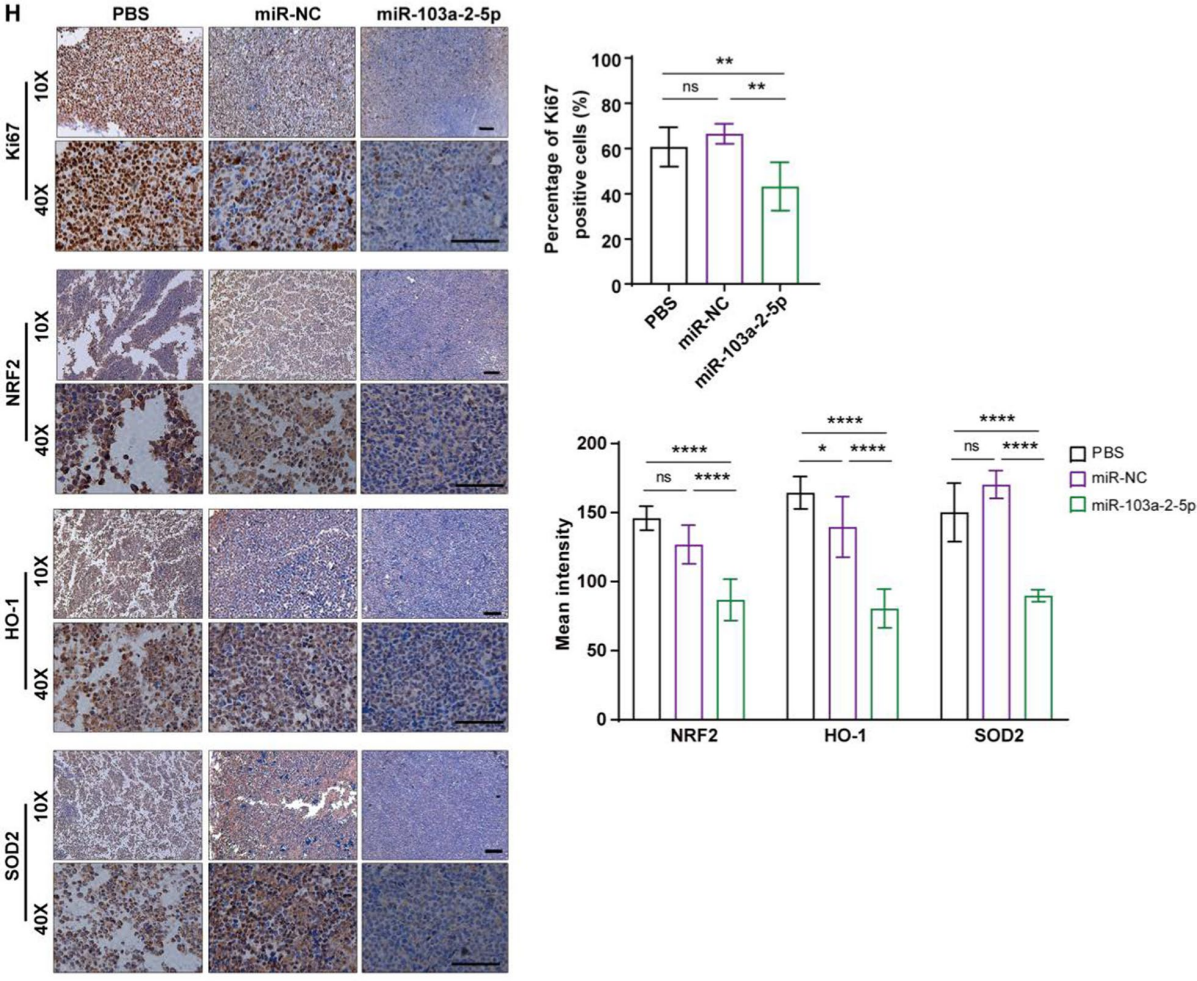
The antitumor activity in vivo. **A** Schematic illustrating the CLPs-miR treatment assays. THP-1 cells were implanted subcutaneously into BALB/c-nu mice (**B**) Growth record curves of subcutaneous tumors in nude mice during the experiment. **C** Representative photographs of subcutaneous tumors were harvested from all treatment groups. **D** Tumors harvested from nude mice were weighed. **E**, **G** The subcutaneous tumors of mice in all groups were removed and ground, and then western blot assays were used to detect the expression of the listed proteins. (**H**) Immunohistochemical analysis of Ki67, NRF2, HO-1, and SOD2. Scare bar: 100 µm. (n = 4; mean ± SD; One-way ANOVA), *P < 0.05, **P < 0.01, ***P < 0.001, ns: no significance, vs. control miRNA (miR-NC) and PBS

The process of animal experiments was shown in Fig. 8A. In nude mouse xenografts, the treatment group with subcutaneous injection of CLPs-miR-103a-2-5p had a slower tumor growth rate than the remaining two control groups (Fig. 8B). When the mice were sacrificed, the tumors were removed from the mice and weighed. The volume and weight of tumors in the CLPs-miR-103a-2-5p group significantly decreased compared with control groups (Fig. 8C, D). Western blot analysis of xenograft tumor issues showed that cycle-related proteins CDK4 and cyclinD1, and apoptotic-related protein Bcl2 expression decreased while that of p21, p53, Bax, and cleaved caspase3 protein increased, suggesting miR-103a-2-5p could affect the cell cycle and apoptosis of AML (Fig. 8E, F). The protein level of the Nrf2/HO-1 signaling pathway, including its downstream molecules SOD2 and NQO1, was significantly reduced in the miR-103a-2-5p group (Fig. 8G). Ki67 staining was performed to observe tumor proliferation after the removal of subcutaneous tumors in mice. The percentage of Ki67-positive cells in the miR-103a-2-5p group was lower than that in the control group. The IHC analysis showed that the NRF2, HO-1, and SOD2 expression in the miR-103a-2-5p group were significantly lower than that in the control group, indicating that this pathway was less activated (Fig. 8H). To better show the effect of miR-103a-2-5p in vivo, we injected GFP-tagged THP-1 into the tail vein. Flow cytometry manifested that CLPs-miR-103a-2-5p could inhibit the propagation of human AML cells in the BM and spleen and increase the survival time of the mice (Additional file 10: Fig. S5A, B). CLPs-miR-103a-2-5p resulted in a reduction in spleen weight (Additional file 10: Fig. S5C). qRT-PCR showed that compared with the PBS group and the miR-NC group, after injecting the miR-103a-2-5p mimic, miR-103a-2-5p expression was observably elevated (Additional file 10: Fig. S5D). All of these results suggested that the miR-103a-2-5p could eventually modulate the proliferation in AML cells by the Nrf2/HO-1 signaling pathway.

### Safety of CLPs-miR-103a-2-5p in vivo

**Fig. 9:**
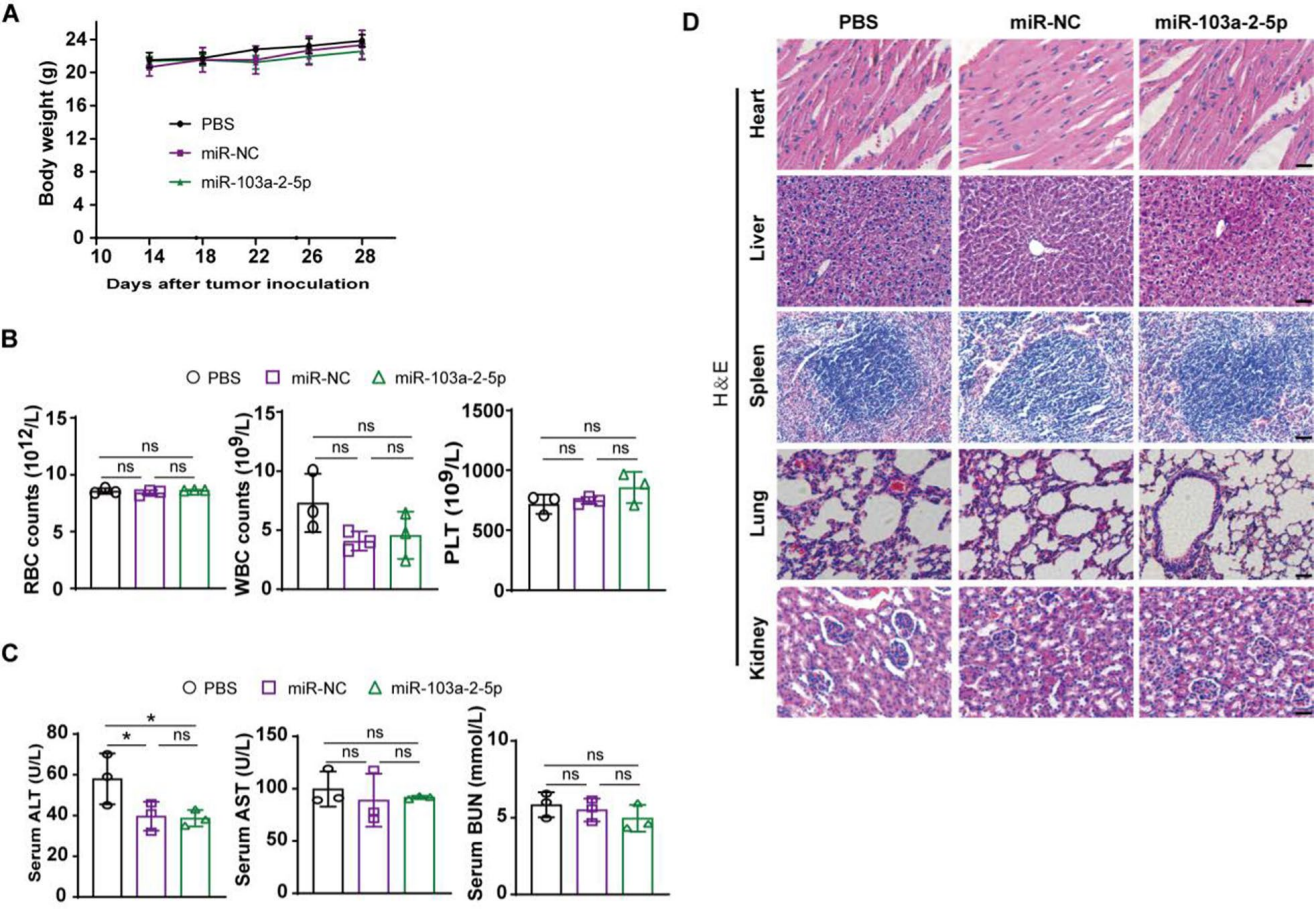
In vivo safety assessment. **A** Body weight change curves of nude mice in all treatment groups during the experiment. **B** Routine blood test of mice after the treatment. **C** Changes in biochemical indicators of liver and kidney function in mice after the treatment. **D** H&E staining of the main organs of nude mice. Scale bar: 100 µm

To assess the safety of the CLPs-miR-103a-2-5p nanoplatform in vivo, we measured and recorded the changes in mouse body weight in all treatment groups throughout the entire trial. The body weight of mice in the three groups exhibited no significant change (Fig. 9A). Furthermore, the routine blood test showed that there was no significant change in the counts of red blood cell (RBC), white blood cell (WBC), and platelet (PLT) of all treatment groups (Fig. 9B; Additional file 10: Fig. S5E). Liver function (serum alanine aminotransferase, ALT, and aspartate aminotransferase, AST) and kidney function (serum urea nitrogen, BUN) were also measured. For subcutaneous xenograft models, only ALT in the PBS group showed a significant difference compared to the other two groups, whereas all other groups did not display a significant difference in liver function and kidney function (Fig. 9C). For the tail vein xenograft model, there was no significant change in liver function and kidney function among three groups (Additional file 10: Fig. S5F). In addition, we further analyzed the histomorphological changes in the main organs of the tumor-bearing mice in various treatment groups. Similarly, all groups did not display tissue injury (Fig. 9D). These results confirmed that CLPs have no hepatotoxicity and nephrotoxicity.

## Discussion

We are entering into a new era where AML patients will have more alternatives for treatment. Even with this progress, there is still a great deal of unmet needs in AML therapy. In the foreseeable future, combination therapies, particularly single or multiple-targeted therapies, will still be the mainstay of AML treatment. Hopefully, immunotherapy might also play a larger role in AML [2].

It has been reported that certain cells of myeloid leukemia, B lymphoid leukemia, and myeloma express LILRB3 [20]. Cell proliferation in human leukemia cell lines is suppressed when LILRB3 expression is inhibited [8]. In addition, LILRB3 might play a role in the negative regulation of immune responses. For instance, blocking LILRB3 promotes the proliferation and differentiation of T helper cells whereas knocking down LILRB3 in macrophages improves phagocytosis and antigen presentation [33]. Wu et al. have demonstrated that the TRAF2–cFLIP–NF-κB signaling axis is the mechanism whereby LILTB3 regulates T-cell antitumor immunological responses [7]. Furthermore, compared to AML patients with low LILRB3 expression, those with high LILRB3 expression had lower survival outcomes according to our clinical data. Above all, it is proposed that LILRB3 is a valuable therapeutic target.

MiRNAs can control a widely distributed network since miRNAs do not require precise base pairing. Furthermore, it is known that miRNAs control hematopoiesis, apoptosis, and cell proliferation, which makes them potential targets for therapeutics and diagnostics. To further elucidate the regulation mechanism of LILRB3 in AML, a bioinformatics website was used to predict and screen the potential targets of LILRB3, and miR-103a-2-5p was found to bind directly to the 3’-UTR of LILRB3. The function of miR-103a-2-5p in tumors has been reported [34]. Several studies implicated the oncogenic role of miR-103a in CRC [35]. It has also been shown that miR-103a could suppress the cell growth, migration, and invasion of gastric cancer [36], and miR-103a-2-5p could target PARP-1 to inhibit colony formation and cell survival [37]. However, the function of miR-103a-2-5p in AML remains unknown to date. Our clinical data suggested a possible prognostic significance and therapeutic target for miR-103a-2-5p in AML. Our research demonstrated that miR-103a-2-5p could regulate the expression of LILRB3, inhibit proliferative capacity, and induce apoptosis of AML cells. The direct binding of miR-103a-2-5p to LILRB3 was confirmed by the luciferase reporter assay, suggesting that the miR-103a-2-5p/LILRB3 pathway may play a role in regulating cell proliferation by modulating the NF-κB pathways. Meanwhile, the miR-103a-2-5p/LILRB3 pathway may inhibit the expression of immunosuppressive receptors while enhancing the CD8 + T cell response.

Different from other antioxidants, HO-1 can be involved in the immunological response, survival and growth, apoptotic response, and metabolism. It has been observed that HO-1, specifically in AML cells, promotes resistance to apoptosis caused by TNFα [38]. Our study showed that HO-1 expression was significantly suppressed in the overexpressing miR-103a-2-5p group, suggesting that HO-1 may be involved in the regulation of apoptosis in tumor cells. Numerous transcription factors are attributed to the regulation of HO-1. Our findings highlighted the transcriptional activation of HO-1 by NRF2, a master regulator of antioxidant transcription known to be involved in leukemogenesis [39]. NRF2 is also a transcription factor that mediates cellular antioxidative stress responses. The Nrf2/HO-1 pathway is an important antioxidant pathway in cells in healthy settings, whose activation can promote GSH synthesis and the expression of various downstream antioxidant proteins, such as HO-1 and SOD2. These changes could remove ROS and protect cells from oxidative damage, exerting anti-inflammatory and anti-cancer effects. However, by regulating NRF2 activity, oxidative stress plays a major role in the pathophysiology of chronic disorders like cancer. Increased NRF2 promotes cell growth and proliferation and inhibits apoptosis in cancer cells to induce cancer progression. As a result, inhibiting NRF2 activity is considered a potential therapeutic approach for preventing cancer. GSH plays an important role in cell processes such as differentiation, proliferation, and apoptosis, which removes excessive ROS and maintains intracellular redox balance [40]. Our data suggest that miR-103a-2-5p overexpression may reduce the ratio of GSH/ROS, leading to excessive intracellular ROS that may promote cell apoptosis and regulate tumor proliferation. Through in-depth study, we found that miR-103a-2-5p overexpression downregulated Nrf2/HO-1 pathway activity, inhibiting intracellular GSH levels and promoting ROS production, thus causing tumor cell apoptosis.

With an increasing interest in miRNA therapeutics, miRNA-based drugs are being innovatively tested in phase II clinical trials for cancer [41]. Examples of these trials include Cobomarsen (anti-miR-155) for T-cell leukemia/lymphoma, TargomiR (miR-16 mimic-based therapy) for mesothelioma [42], and Miravirsen (anti-miR-122) for hepatitis C infection [43]. Since miRNA mimics are quickly degraded and cleared, tissue-specific delivery is challenging [44]. Present studies have shown that LNPs can be used as effective RNA-delivering vehicles targeting tumor cells, which can maximize therapeutic efficiency while minimizing systemic side effects [45–47]. We designed liposome packaged miRNA system CLPs, characterized by a nanoparticle size to protect miRNAs from degradation and increase the stability of miRNAs in circulation. Furthermore, by adjusting the charge ratio of DOTAP to miRNAs to 4:1, we used CLPs to deliver miR-103a-2-5p into AML cells and confirmed the inhibitory effect of CLPs- miR-103a-2-5p on AML cells in vivo.

## Conclusion

**Fig. 10:**
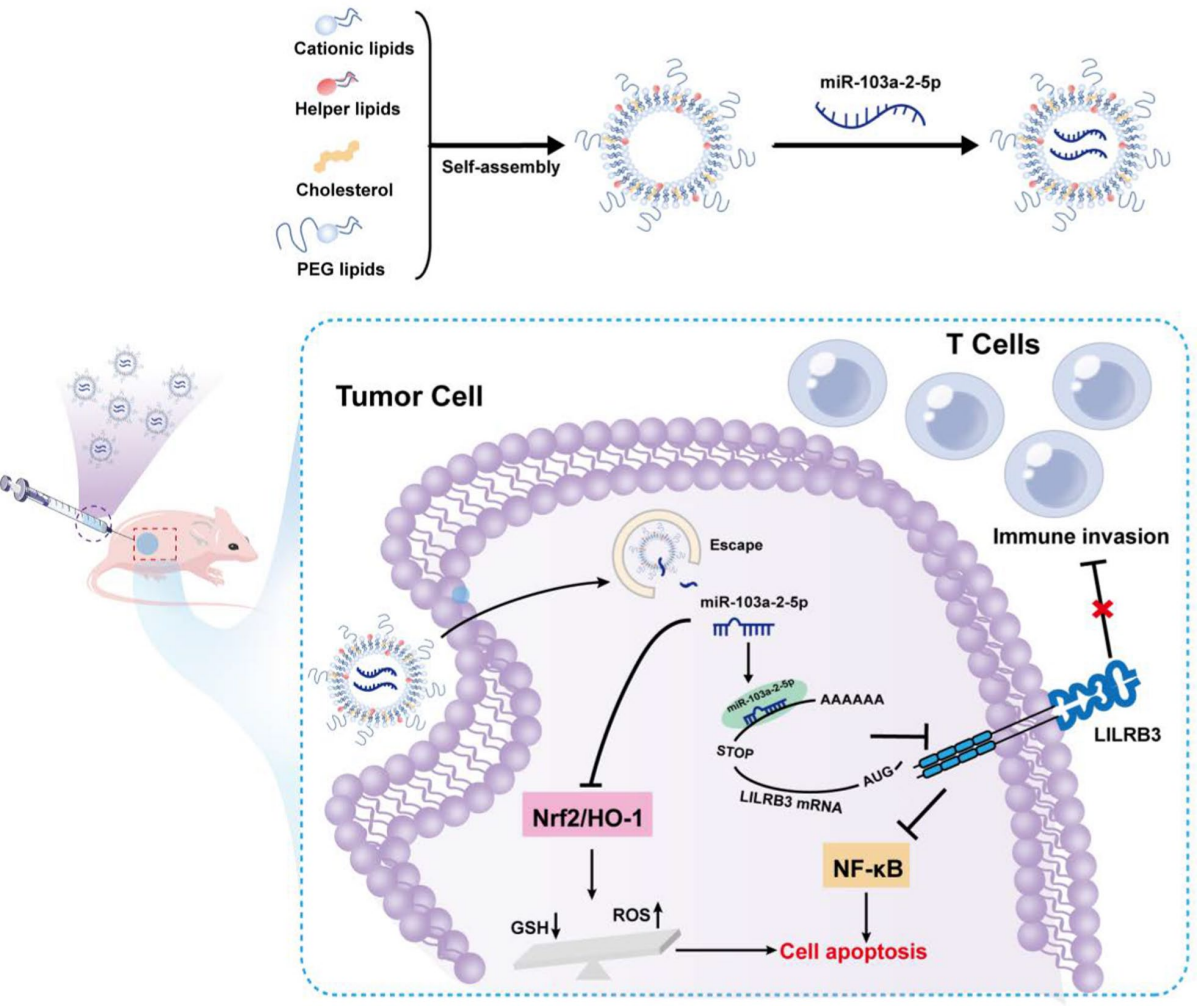
Diagram of the formative process and mechanism of action of CLPs-miR-103a-2-5p. CLPs-miR-103a-2-5p are synthesized by the thin-film hydration method. Firstly, cationic liposomes are synthesized by assembly with DOTAP, DSPC, cholesterol, and DSPE-PEG. Then, liposomes and miR-103a-2-5p are mixed by oscillation. MiR-103a-2-5p suppresses the expression of LILRB3, resulting in the promotion of AML cell apoptosis and T-cell antitumor immune responses. Meanwhile, miR-103a-2-5p downregulates Nrf2/HO-1 pathway activity, inhibiting intracellular GSH levels and promoting ROS production, thereby causing tumor cell apoptosis

In conclusion, the current findings demonstrate that miR-103a-2-5p serves as a tumor suppressor targeting LILRB3 to inhibit proliferation and induce apoptosis in AML cells. It downregulates Nrf2/HO-1 pathway activity, inhibiting intracellular GSH levels and promoting ROS production, thereby causing tumor cell apoptosis. Therefore, targeting miR-103a-2-5p could be a potential immunotherapeutic strategy in treating LILRB3-altered AML. Besides, our findings indicated that miR-103a-2-5p may enhance the CD8 + T cell response by suppressing LILRB3 expression (Fig. 10). Our results also provide a theoretical foundation for the development of new immunotherapy for AML.

### Supplementary Information

**Additional file 1.** MiRNAs predicted by three databases.

**Additional file 2.** Clinical characteristics of AML samples used.

**Additional file 3.** Plaimd sequence and miRNA sequence.

**Additional file 4.** PCR primer sequences utilized in this study.

**Additional file 5.** HLA typing of healthy donors and AML cells.

**Additional file 6.** Four candidate miRNAs expression in AML cells.

**Additional file 7.** The inhibitory effect of miR-103a-2-5p on the migration and colony formation of AML cells.

**Additional file 8.** MiR-103a-2-5p enhances CD8+ T cells survival and reduces inhibitory receptor generation.

**Additional file 9.** The mRNA expression of genes related to cell apoptosis, cell cycle, and antioxidation.

**Additional file 10.** The influence of miR-103a-2-5p in the AML tail vein mouse model.

## Data Availability

Data will be made available on reasonable request.
