# Supplementary material for: CLPs-miR-103a-2-5p inhibits proliferation and promotes cell apoptosis in AML cells by targeting LILRB3 and Nrf2/HO-1 axis, regulating CD8 + T cell response
Source: J Transl Med. 2024 Mar 14;22:278. doi: 10.1186/s12967-024-05070-5 (PMC10938737; doi:10.1186/s12967-024-05070-5)
Supplement: Supplementary file 1 — Additional file 1. MiRNAs predicted by three databases. [file 12967_2024_5070_MOESM1_ESM.docx]

**Table S1. MiRNAs predicted by three databases.**

| **miRDA** | **TargetScan** | **miRWalk** |
| --- | --- | --- |
| hsa-miR-103a-1-5p  hsa-miR-103a-2-5p  hsa-miR-382-3p  hsa-miR-504-3p  hsa-miR-1208  hsa-miR-5692c  hsa-miR-5692b  hsa-miR-5702  hsa-miR-4279  hsa-miR-581  hsa-miR-543  hsa-miR-6778-3p  hsa-miR-6071  hsa-miR-7107-3p  hsa-miR-6773-3p  hsa-miR-6753-3p  hsa-miR-367-5p  hsa-miR-8077  hsa-miR-8081  hsa-miR-512-3p  hsa-miR-4445-5p  hsa-miR-1304-3p  hsa-miR-122-5p  hsa-miR-1200 | hsa-miR-7704  hsa-miR-615-5p  hsa-miR-6742-5p  hsa-miR-491-5p  hsa-miR-6796-5p  hsa-miR-4472  hsa-miR-4447  hsa-miR-3151-5p  hsa-miR-574-5p  hsa-miR-4658  hsa-miR-6790-5p  hsa-miR-3659  hsa-miR-8077  hsa-miR-4663  hsa-miR-7160-5p  hsa-miR-93-3p  hsa-miR-6071  hsa-miR-3692-5p  hsa-miR-6828-3p  hsa-miR-1237-3p  hsa-miR-1248  hsa-miR-6868-3p  hsa-miR-924  hsa-miR-627-5p  hsa-miR-4324  hsa-miR-544b  hsa-miR-1200  hsa-miR-6838-3p  hsa-miR-1910-5p  hsa-miR-4274  hsa-miR-4291  hsa-miR-214-3p  hsa-miR-761  hsa-miR-3619-5p  hsa-miR-922  hsa-miR-5581-5p  hsa-miR-4297  hsa-miR-6736-3p  hsa-miR-4660  hsa-miR-486-3p  hsa-miR-4488  hsa-miR-1237-5p  hsa-miR-4697-5p  hsa-miR-6848-5p  hsa-miR-6846-5p  hsa-miR-637  hsa-miR-181a-2-3p  hsa-miR-1273g-3p  hsa-miR-6509-3p  hsa-miR-7978  hsa-miR-653-3p  hsa-miR-181b-3p  hsa-miR-181b-2-3p  hsa-miR-4420  hsa-miR-6855-5p  hsa-miR-3170  hsa-miR-193b-5p  hsa-miR-7155-5p  hsa-miR-3929  hsa-miR-4478  hsa-miR-4419b  hsa-miR-4645-5p  hsa-miR-4673  hsa-miR-615-5p  hsa-miR-6782-5p  hsa-miR-6824-5p  hsa-miR-4278  hsa-miR-6789-5p  hsa-miR-1258  hsa-miR-3152-3p  hsa-miR-7107-3p  hsa-miR-6753-3p  hsa-miR-6503-5p  hsa-miR-4527  hsa-miR-6072  hsa-miR-6891-3p  hsa-miR-4279  hsa-miR-7977  hsa-miR-125b-2-3p  hsa-miR-188-3p  hsa-miR-4433a-5p  hsa-miR-5588-3p  hsa-miR-125a-3p  hsa-miR-3934-5p  hsa-miR-764  hsa-miR-5702  hsa-miR-937-5p  hsa-miR-8081  hsa-miR-627-5p  hsa-miR-92b-5p  hsa-miR-4698  hsa-miR-367-5p  hsa-miR-1267  hsa-miR-1253  hsa-miR-6770-5p  hsa-miR-580-3p  hsa-miR-4445-5p  hsa-miR-508-3p  hsa-miR-4782-3p  hsa-miR-219a-5p  hsa-miR-6766-3p  hsa-miR-6750-3p  hsa-miR-4764-3p  hsa-miR-4477a  hsa-miR-129-5p  hsa-miR-129-5p  hsa-miR-941  hsa-miR-140-3p.2  hsa-miR-624-5p  hsa-miR-1911-5p  hsa-miR-4324  hsa-miR-544b  hsa-miR-1200  hsa-miR-3653-5p  hsa-miR-660-3p  hsa-miR-6829-3p  hsa-miR-6791-3p  hsa-miR-512-5p  hsa-miR-4286  hsa-miR-6749-3p  hsa-miR-6830-5p  hsa-miR-561-5p  hsa-miR-124-5p  hsa-miR-581  hsa-miR-578  hsa-miR-761  hsa-miR-214-3p  hsa-miR-3619-5p  hsa-miR-922  hsa-miR-4291  hsa-miR-7156-3p  hsa-miR-1184  hsa-miR-4418  hsa-miR-509-3-5p  hsa-miR-509-5p  hsa-miR-6890-3p  hsa-miR-1304-3p  hsa-miR-6854-5p  hsa-miR-7705  hsa-miR-103b  hsa-miR-891a-3p  hsa-miR-5693  hsa-miR-6499-3p  hsa-miR-143-5p  hsa-miR-504-3p  hsa-miR-122-5p  hsa-miR-3135b  hsa-miR-3652  hsa-miR-4430  hsa-miR-4438  hsa-miR-6768-5p  hsa-miR-377-3p  hsa-miR-6773-3p  hsa-miR-215-3p  hsa-miR-6812-3p  hsa-miR-624-3p  hsa-miR-892c-3p  hsa-miR-452-5p  hsa-miR-4676-3p  hsa-miR-1208  hsa-miR-6760-3p  hsa-miR-4420  hsa-miR-653-3p  hsa-miR-181b-2-3p  hsa-miR-181b-3p  hsa-miR-181c-5p  hsa-miR-181a-5p  hsa-miR-181b-5p  hsa-miR-181d-5p  hsa-miR-4262  hsa-miR-543  hsa-miR-4328  hsa-miR-382-3p  hsa-miR-323a-3p  hsa-miR-4699-3p  hsa-miR-5692c  hsa-miR-5692b  hsa-miR-369-3p  hsa-miR-374a-5p  hsa-miR-374b-5p  hsa-miR-568  hsa-miR-3171  hsa-miR-148a-5p  hsa-miR-4446-5p  hsa-miR-4775  hsa-miR-590-3p  hsa-miR-103a-2-5p  hsa-miR-491-3p  hsa-miR-1292-3p  hsa-miR-6807-5p  hsa-miR-3186-5p  hsa-miR-1295b-3p  hsa-miR-5186  hsa-miR-8077  hsa-miR-4663  hsa-miR-7160-5p  hsa-miR-512-3p  hsa-miR-520c-3p  hsa-miR-372-3p  hsa-miR-373-3p  hsa-miR-520b  hsa-miR-302a-3p  hsa-miR-302b-3p  hsa-miR-302d-3p  hsa-miR-302c-3p.1  hsa-miR-520e  hsa-miR-520a-3p  hsa-miR-520d-3p  hsa-miR-302e  hsa-miR-20a-5p  hsa-miR-106b-5p  hsa-miR-519d-3p  hsa-miR-20b-5p  hsa-miR-93-5p  hsa-miR-17-5p  hsa-miR-106a-5p  hsa-miR-526b-3p  hsa-miR-520g-3p  hsa-miR-520h  hsa-miR-186-3p  hsa-miR-150-5p  hsa-miR-6778-3p  hsa-miR-4711-5p  hsa-miR-4454  hsa-miR-433-3p  hsa-miR-6790-3p  hsa-miR-6821-3p  hsa-miR-378a-5p  hsa-miR-3653-5p  hsa-miR-1976  hsa-miR-6843-3p  hsa-miR-6848-3p  hsa-miR-2392  hsa-miR-6858-3p  hsa-miR-4676-5p  hsa-miR-575  hsa-miR-4662a-5p  hsa-miR-3152-3p  hsa-miR-5096  hsa-miR-193b-5p  hsa-miR-483-5p  hsa-miR-6832-5p  hsa-miR-411-5p.2  hsa-miR-411-5p.1  hsa-miR-3149  hsa-miR-129-5p  hsa-miR-4775  hsa-miR-590-3p  hsa-miR-4735-5p  hsa-miR-6788-3p  hsa-miR-3622a-5p  hsa-miR-4423-3p  hsa-miR-5582-5p  hsa-miR-1295b-3p  hsa-miR-625-3p  hsa-miR-365b-5p  hsa-miR-365a-5p  hsa-miR-3199  hsa-miR-8052  hsa-miR-6869-5p  hsa-miR-4324  hsa-miR-544b  hsa-miR-1200  hsa-miR-3653-5p  hsa-miR-660-3p  hsa-miR-6829-3p  hsa-miR-6791-3p  hsa-miR-6747-3p  hsa-miR-6727-3p  hsa-miR-4722-3p  hsa-miR-1976  hsa-miR-3653-5p  hsa-miR-4279  hsa-miR-6847-3p  hsa-miR-4437  hsa-miR-4532  hsa-miR-1247-3p  hsa-miR-6778-3p  hsa-miR-6836-3p  hsa-miR-6791-3p  hsa-miR-6829-3p  hsa-miR-5001-3p  hsa-miR-3194-3p  hsa-miR-6796-3p  hsa-miR-2276-3p  hsa-miR-6890-3p  hsa-miR-1304-3p  hsa-miR-6736-3p  hsa-miR-6787-3p  hsa-miR-4695-3p  hsa-miR-4466  hsa-miR-675-5p  hsa-miR-6499-3p  hsa-miR-143-5p  hsa-miR-504-3p  hsa-miR-122-5p  hsa-miR-3135b  hsa-miR-3652  hsa-miR-4430  hsa-miR-3664-5p  hsa-miR-6794-3p  hsa-miR-4639-3p  hsa-miR-1304-3p  hsa-miR-1285-5p  hsa-miR-216a-5p  hsa-miR-103a-2-5p  hsa-miR-487a-5p  hsa-miR-487b-5p | hsa-miR-892c-3p  hsa-miR-1470  hsa-miR-509-5p  hsa-miR-600  hsa-miR-4800-3p  hsa-miR-6078  hsa-miR-892c-3p  hsa-miR-6869-3p  hsa-miR-6073  hsa-miR-146a-3p  hsa-miR-572  hsa-miR-572  hsa-miR-6726-3p  hsa-miR-148a-5p  hsa-miR-376a-5p  hsa-miR-552-3p  hsa-miR-601  hsa-miR-1224-3p  hsa-miR-892a  hsa-miR-873-3p  hsa-miR-924  hsa-miR-1231  hsa-miR-1237-3p  hsa-miR-4274  hsa-miR-4277  hsa-miR-3663-5p  hsa-miR-3679-3p  hsa-miR-3691-3p  hsa-miR-4657  hsa-miR-4697-5p  hsa-miR-4711-3p  hsa-miR-4721  hsa-miR-4745-3p  hsa-miR-5572  hsa-miR-5588-3p  hsa-miR-5702  hsa-miR-6068  hsa-miR-6842-5p  hsa-miR-8077  hsa-miR-6529-3p  hsa-miR-208a-5p  hsa-miR-214-5p  hsa-let-7i-3p  hsa-miR-106b-3p  hsa-miR-148b-5p  hsa-miR-501-5p  hsa-miR-558  hsa-miR-550a-3p  hsa-miR-636  hsa-miR-646  hsa-miR-662  hsa-miR-1296-3p  hsa-miR-670-5p  hsa-miR-924  hsa-miR-1539  hsa-miR-1911-5p  hsa-miR-3184-3p  hsa-miR-3186-5p  hsa-miR-3616-3p  hsa-miR-3921  hsa-miR-4657  hsa-miR-4711-3p  hsa-miR-2467-5p  hsa-miR-4796-5p  hsa-miR-548at-5p  hsa-miR-5708  hsa-miR-6734-3p  hsa-miR-6759-3p  hsa-miR-6768-3p  hsa-miR-6774-3p  hsa-miR-6833-3p  hsa-miR-6849-3p  hsa-miR-6858-3p  hsa-miR-6869-3p  hsa-miR-6869-3p  hsa-miR-6885-5p  hsa-miR-6887-3p  hsa-miR-7153-3p  hsa-miR-7162-5p  hsa-miR-8071  hsa-miR-8078  hsa-miR-9901  hsa-miR-10401-3p  hsa-miR-6529-3p  hsa-miR-9851-5p  hsa-let-7a-2-3p  hsa-miR-26b-3p  hsa-miR-103a-2-5p  hsa-miR-208a-5p  hsa-miR-129-5p  hsa-miR-148a-5p  hsa-miR-214-5p  hsa-miR-222-3p  hsa-let-7i-3p  hsa-miR-124-3p  hsa-miR-34c-5p  hsa-miR-34c-5p  hsa-miR-376a-5p  hsa-miR-148b-5p  hsa-miR-346  hsa-miR-425-3p  hsa-miR-329-3p  hsa-miR-494-5p  hsa-miR-496  hsa-miR-512-5p  hsa-miR-501-5p  hsa-miR-504-5p  hsa-miR-504-3p  hsa-miR-552-3p  hsa-miR-92b-5p  hsa-miR-92b-3p  hsa-miR-558  hsa-miR-578  hsa-miR-587  hsa-miR-550a-3p  hsa-miR-601  hsa-miR-618  hsa-miR-619-5p  hsa-miR-619-5p  hsa-miR-629-3p  hsa-miR-636  hsa-miR-645  hsa-miR-646  hsa-miR-654-3p  hsa-miR-659-3p  hsa-miR-668-3p  hsa-miR-1224-3p  hsa-miR-670-5p  hsa-miR-670-5p  hsa-miR-770-5p  hsa-miR-892a  hsa-miR-885-3p  hsa-miR-873-3p  hsa-miR-924  hsa-miR-935  hsa-miR-1180-5p  hsa-miR-1183  hsa-miR-1229-3p  hsa-miR-1231  hsa-miR-1237-3p  hsa-miR-1285-5p  hsa-miR-1287-5p  hsa-miR-1284  hsa-miR-1470  hsa-miR-1470  hsa-miR-1909-5p  hsa-miR-1910-5p  hsa-miR-1911-5p  hsa-miR-1911-3p  hsa-miR-1914-5p  hsa-miR-2277-5p  hsa-miR-718  hsa-miR-3120-5p  hsa-miR-3130-5p  hsa-miR-3169  hsa-miR-3177-5p  hsa-miR-3184-3p  hsa-miR-3186-5p  hsa-miR-3191-5p  hsa-miR-4258  hsa-miR-4252  hsa-miR-4265  hsa-miR-4269  hsa-miR-4277  hsa-miR-4284  hsa-miR-3616-3p  hsa-miR-3619-5p  hsa-miR-3663-5p  hsa-miR-3664-5p  hsa-miR-3667-3p  hsa-miR-3677-3p  hsa-miR-3679-3p  hsa-miR-3691-3p  hsa-miR-3150b-5p  hsa-miR-3921  hsa-miR-3936  hsa-miR-550b-3p  hsa-miR-378g  hsa-miR-4430  hsa-miR-4430  hsa-miR-4435  hsa-miR-4443  hsa-miR-4454  hsa-miR-4483  hsa-miR-4657  hsa-miR-4674  hsa-miR-4691-3p  hsa-miR-4695-3p  hsa-miR-4697-5p  hsa-miR-4711-3p  hsa-miR-4713-5p  hsa-miR-4714-5p  hsa-miR-4715-5p  hsa-miR-4717-5p  hsa-miR-4721  hsa-miR-4724-3p  hsa-miR-4727-5p  hsa-miR-4740-5p  hsa-miR-4740-3p  hsa-miR-4742-3p  hsa-miR-4745-3p  hsa-miR-4753-3p  hsa-miR-4772-3p  hsa-miR-4781-3p  hsa-miR-2467-5p  hsa-miR-4797-5p  hsa-miR-5187-3p  hsa-miR-5196-3p  hsa-miR-5581-3p  hsa-miR-5588-3p  hsa-miR-5685  hsa-miR-5698  hsa-miR-5702  hsa-miR-5708  hsa-miR-1199-3p  hsa-miR-6068  hsa-miR-6075  hsa-miR-6499-5p  hsa-miR-6507-3p  hsa-miR-6509-3p  hsa-miR-6731-3p  hsa-miR-6734-3p  hsa-miR-6738-3p  hsa-miR-6749-3p  hsa-miR-6750-3p  hsa-miR-6754-3p  hsa-miR-6757-5p  hsa-miR-6759-3p  hsa-miR-6772-3p  hsa-miR-6774-3p  hsa-miR-6783-3p  hsa-miR-6784-3p  hsa-miR-6789-3p  hsa-miR-6789-3p  hsa-miR-6791-3p  hsa-miR-6799-3p  hsa-miR-6800-3p  hsa-miR-6809-3p  hsa-miR-6814-3p  hsa-miR-6817-5p  hsa-miR-6821-3p  hsa-miR-6833-3p  hsa-miR-6834-3p  hsa-miR-6842-5p  hsa-miR-6849-3p  hsa-miR-6858-3p  hsa-miR-6868-3p  hsa-miR-6869-3p  hsa-miR-6872-3p  hsa-miR-6875-3p  hsa-miR-6879-3p  hsa-miR-6881-3p  hsa-miR-6882-3p  hsa-miR-6885-5p  hsa-miR-6886-3p  hsa-miR-6895-3p  hsa-miR-7109-3p  hsa-miR-7151-5p  hsa-miR-7153-3p  hsa-miR-7161-3p  hsa-miR-8052  hsa-miR-8071  hsa-miR-8077  hsa-miR-9899  hsa-miR-9901  hsa-miR-10401-3p  hsa-miR-6529-3p  hsa-miR-9851-5p  hsa-miR-12119 |
