# Supplementary material for: CLPs-miR-103a-2-5p inhibits proliferation and promotes cell apoptosis in AML cells by targeting LILRB3 and Nrf2/HO-1 axis, regulating CD8 + T cell response
Source: J Transl Med. 2024 Mar 14;22:278. doi: 10.1186/s12967-024-05070-5 (PMC10938737; doi:10.1186/s12967-024-05070-5)
Supplement: Supplementary file 2 — Additional file 2. Clinical characteristics of AML samples used. [file 12967_2024_5070_MOESM2_ESM.docx]

**Table S2. Clinical characteristics of AML samples used. LILRB3 mRNA expression and relative miR-103a-2-5p expression levels**

| **Number** | **Age** | **Gender** | **FAB**  **Classification** | **%Blasts** | **Gene Mutation** | **LILRB3 level**  **2^-ΔΔCT** | **MiR-103a-2-5p level 2^-ΔΔCT** |
| --- | --- | --- | --- | --- | --- | --- | --- |
| **AML#1** | **76** | **Female** | **M4** | **92.2** | **FLT3-ITD,CEBPA,NPM1** | **7.804** | **0.004** |
| **AML#2** | **24** | **Female** | **M5** | **59.2** | **CR1,FLT3-ITD,NPM1** | **7.724** | **0.005** |
| **AML#3** | **38** | **Female** | **M4** | **41.1** | **FLT3-TKD,DNMT3A,NPM1** | **7.664** | **0.034** |
| **AML#4** | **67** | **Female** | **M4** | **19.7** | **WT1** | **6.389** | **0.011** |
| **AML#5** | **27** | **Female** | **M4** | **75.0** | **CEBPA,TET2,NF1** | **5.494** | **0.178** |
| **AML#6** | **34** | **Female** | **M5** | **87.5** | **DNMT3A,NPM2,FLT3-ITD** | **4.631** | **0.070** |
| **AML#7** | **27** | **Male** | **M4** | **72.6** | **FLT3-ITD,WT1,RUNX1** | **4.585** | **1.024** |
| **AML#8** | **36** | **Female** | **M5** | **75.0** | **MYH11+,FLT3-ITD** | **4.576** | **0.004** |
| **AML#9** | **16** | **Female** | **M4** | **81.5** | **WT1,ASXL1,SETD2** | **4.454** | **1.566** |
| **AML#10** | **26** | **Male** | **M1** | **83.1** | **CEBPA,NRAS,WT1** | **4.416** | **0.105** |
| **AML#11** | **35** | **Female** | **M4** | **74.0** | **WT1,FLT3-TKD,NF1** | **4.197** | **0.010** |
| **AML#12** | **26** | **Male** | **M5b** | **30.5** | **NPM-MLF1,FLT3-ITD** | **3.362** | **0.581** |
| **AML#13** | **45** | **Female** | **M1** | **85.5** | **CEBPA** | **3.338** | **0.136** |
| **AML#14** | **21** | **Female** | **M4** | **70.0** | **CEBPA,CSF3R,NRAS** | **3.291** | **0.009** |
| **AML#15** | **55** | **Female** | **M2** | **31.0** | **WT1,PTPN,STAF** | **3.178** | **0.027** |
| **AML#16** | **30** | **Male** | **M2** | **87.5** | **WT1,IKZF1,NARS** | **3.112** | **0.490** |
| **AML#17** | **73** | **Female** | **M4** | **45.5** | **ASXL1,BCOR,DNMT3A** | **3.038** | **0.071** |
| **AML#18** | **57** | **Male** | **M2** | **80** | **MLL-PTD,WT1** | **2.807** | **0.039** |
| **AML#19** | **65** | **Female** | **M5b** | **20.5** | **WT1** | **2.805** | **0.028** |
| **AML#20** | **24** | **Female** | **M1** | **68.5** | **WT1,TET2,STEG2** | **2.735** | **2.763** |
| **AML#21** | **41** | **Female** | **M2** | **79.0** | **MLL-PTD,FLT3-ITD,IDH2** | **2.704** | **0.017** |
| **AML#22** | **59** | **Male** | **M5** | **70.0** | **MLL-PTD,ASXL1,DNMT3A** | **2.174** | **2.004** |
| **AML#23** | **45** | **Female** | **M3** | **46.7** | **WT1,NPM1,DNMT3A** | **2.161** | **0.011** |
| **AML#24** | **68** | **Male** | **M1** | **56.5** | **NRAS,TET2,CEBPA** | **2.087** | **0.014** |
| **AML#25** | **31** | **Male** | **M2** | **46.0** | **WT1,NPM1,NRAS** | **2.040** | **0.663** |
| **AML#26** | **53** | **Male** | **M2** | **68.5** | **GATA2,IKZF1,PHF6** | **1.249** | **0.520** |
| **AML#27** | **54** | **Female** | **M2** | **27.5** | **CSF3R,RUNX1,ETO** | **0.961** | **2.918** |
| **AML#28** | **29** | **Male** | **M2** | **73.0** | **ETO,CSF3R** | **0.961** | **3.014** |
| **AML#29** | **60** | **Female** | **M2** | **41.9** | **WT1,CEBPA,DNMT3A** | **0.940** | **3.330** |
| **AML#30** | **27** | **Male** | **M2** | **55.5** | **DHX15,EZH2,SMC3** | **0.419** | **1.206** |

The enrolled patients were confirmed with AML in the light of the classification criteria of French, American, Britain (FAB), the World Health Organization (WHO), and the immunophenotypic and cytogenetic analysis.
