# Supplementary material for: CLPs-miR-103a-2-5p inhibits proliferation and promotes cell apoptosis in AML cells by targeting LILRB3 and Nrf2/HO-1 axis, regulating CD8 + T cell response
Source: J Transl Med. 2024 Mar 14;22:278. doi: 10.1186/s12967-024-05070-5 (PMC10938737; doi:10.1186/s12967-024-05070-5)
Supplement: Supplementary file 3 — Additional file 3. Plaimd sequence and miRNA sequence. [file 12967_2024_5070_MOESM3_ESM.docx]

**Table S3. Plaimd sequence and miRNA sequence**

**Sequences of miRNA mimics**

| **Gene name** | **Sense** |
| --- | --- |
| **hsa-miR-103a-2-5p** | **5’-AGCUUCUUUACAGUGCUGCCUUG-3’** |
| **hsa-miR-504-3p** | **5’-GGGAGUGCAGGGCAGGGUUUC-3’** |
| **hsa-miR-5702** | **5’-UGAGUCAGCAACAUAUCCCAUG-3’** |
| **hsa-miR-8077** | **5’-GGCUGAGUGGGGUUCUGACUCC-3’** |

**Sequences of plaimd**

| **LILRB3-3'UTR**  **-WT** | **CTCGAGCCCGGGGGGTACGCAGACCCCACACTCAGCAGAAGGAGACTCAGGACTGCTGAAGGCACGGGAGCTGCCCCCAGTGGACACCAGTGAACCCCAGTCAGCCTGGACCCCTAACACAGACCATGAGGAGACGCTGGGAACTTGTGGGACTCACCTGACTCAAAGATGACTAATATCGTCCCATTTTGGAAATAAAGCAACAGACTTCTCAACAATCAATGAGTTAATAACAAAAAAACAAAAAACAAAAACAGACGTAAAGGCCGGGTGTGGTACTCAGGAGGCTGAGTGGGGAGGATTCCTTGAACACAAGAAGTTAAGGCTGCTGAGGCTGCAGTGAGCTATGACTGTGCCACTGCACTCCAGCCTGTGTGACAGAGCGAGACCTTGTCTCTAAAAAAAAAAACAGTGAATGTTTTAAACTGAATGATAATGTAAATATTATACATCGAACTTATGACATGGGAAAATTAAGAAGCATAAATAGGCCGGGCGCGGTGGCTCACGCCTATAATCTCAGCACTTTGGGAGGCTGATGCGGGCGGATCATGAGGTCAGGAGATCGAGACCATCCTGGCTAACACGGTGAAACCCCGTCTCTACTAAAAATACAAAAAAATTAGCCGGGCGTGGTGGCGAGTGCCTATAGTCCCAGCTACTCAGGAGGCTGAGGCAGGAGAATGGCATGAGCCCGGGAGGCAGAGCTTGCAGTGAGCTGAGATCGCACCACTGCACTCCAGCCTGGGCGACAGAGTGAGATTCCGTCTCGAAAAAAAAAAAAAAAGAAAGAAAAAAAATAAAAAAGAAGCATAACCAGGGTCGAC** |
| --- | --- |
| **LILRB3-3'UTR**  **-MUT** | **CTCGAGCTAAAAATACAAAAAAATTAGCCGGGCGTGGTGGCGAGTGCCTATAGTCCCAGCTACTCAGGAGGCTGAGGCAGGAGAATGGCATGAGCCCGGGAGGCAGAGCTTGCAGTGAGCTGAGATCGCACCACTGCACTCCAGCCTGGGCGACAGAGTGAGATTCCGTCTCGAAAAAAAAAAAAAAAGAAAGAAAAAAAATAAAATTCTTCGTTAACCAGGGTCGAC** |
