# Supplementary material for: CLPs-miR-103a-2-5p inhibits proliferation and promotes cell apoptosis in AML cells by targeting LILRB3 and Nrf2/HO-1 axis, regulating CD8 + T cell response
Source: J Transl Med. 2024 Mar 14;22:278. doi: 10.1186/s12967-024-05070-5 (PMC10938737; doi:10.1186/s12967-024-05070-5)
Supplement: Supplementary file 4 — Additional file 4. PCR primer sequences utilized in this study. [file 12967_2024_5070_MOESM4_ESM.docx]

**Table S4. PCR primer sequences utilized in this study.**

| **Gene name** | **Stem-loop primer** |
| --- | --- |
| **hsa-miR-103a-2-5p** | **GTCGTATCCAGTGCAGGGTCCGAGGTATTCGCACTGGATACGACCAAGGC** |
| **hsa-miR-504-3p** | **GTCGTATCCAGTGCAGGGTCCGAGGTATTCGCACTGGATACGACGAAACC** |
| **hsa-miR-5702** | **GTCGTATCCAGTGCAGGGTCCGAGGTATTCGCACTGGATACGACCATGGG** |
| **hsa-miR-8077** | **GTCGTATCCAGTGCAGGGTCCGAGGTATTCGCACTGGATACGACGGAGTC** |

| **species** | **qRT-PCR** | **Forward 5’-3’** | **Reverse 5’-3’** |
| --- | --- | --- | --- |
| **Human** | **miR-103a-2-5p** | **CGCGAGCTTCTTTACAGTGCT** | **AGTGCAGGGTCCGAGGTATT** |
| **Human** | **miR-8077** | **CGGGCTGAGTGGGGTTCT** | **AGTGCAGGGTCCGAGGTATT** |
| **Human** | **miR-504a-3p** | **CGGGGAGTGCAGGGCAG** | **AGTGCAGGGTCCGAGGTATT** |
| **Human** | **miR-1303** | **CGCGTTTAGAGACGGGGTCT** | **AGTGCAGGGTCCGAGGTATT** |
| **Human** | **miR-5702** | **GCGCGTGAGTCAGCAACATAT** | **AGTGCAGGGTCCGAGGTATT** |
| **Human** | **U6** | **CTCGCTTCGGCAGCACA** | **AACGCTTCACGAATTTGCGT** |
| **Human** | **LILRB3** | **TGCACAGCTTGACCCTTAGAC** | **TGGCCAGAGTGGCGTAGATG** |
| **Human** | **BCL2** | **ATAACGGAGGCTGGGTAGGT** | **TTTATTTCGCCGGCTCCACA** |
| **Human** | **BAX** | **CCCCCGAGAGGTCTTTTTCC** | **TGTCCAGCCCATGATGGTTC** |
| **Human** | **CDK4** | **GTGTATGGGGCCGTAGGAAC** | **CCATAGGCACCGACACCAAT** |
| **Human** | **CyclinD1** | **TCCCACTCCTACGATACGCT** | **TTGAACCTGGACGTGAGCTG** |
| **Human** | **P53** | **ATGATGTGAGTGCTCCCGTG** | **CTCGGAAATTCCCTTGCCCT** |
| **Human** | **p21** | **CTGCCGAAGTCAGTTCCTTG** | **ACCTGTGAACGCAGCACAC** |
| **Human** | **GAPDH** | **GGAGCGAGATCCCTCCAAAAT** | **GGCTGTTGTCATACTTCTCATGG** |
| **Human** | **HO-1** | **TCCTGGCTCAGCCTCAAATG** | **CGTTAAACACCTCCCTCCCC** |
| **Human** | **NRF2** | **CAGCTTTTGGCGCAGACATT** | **GACTGGGCTCTCGATGTGAC** |
| **Human** | **SOD2** | **GGAAGCCATCAAACGTGACTT** | **CCCGTTTCCTTATTGAAACCAAGC** |
| **Human** | **NOQ1** | **AGGATGGAAGAAACGCCTGG** | **TCAGTTGGGATGGACTTGCC** |
