# Supplementary material for: CLPs-miR-103a-2-5p inhibits proliferation and promotes cell apoptosis in AML cells by targeting LILRB3 and Nrf2/HO-1 axis, regulating CD8 + T cell response
Source: J Transl Med. 2024 Mar 14;22:278. doi: 10.1186/s12967-024-05070-5 (PMC10938737; doi:10.1186/s12967-024-05070-5)
Supplement: Supplementary file 5 — Additional file 5. HLA typing of healthy donors and AML cells. [file 12967_2024_5070_MOESM5_ESM.docx]

**Table S5.** HLA typing of healthy donors and AML cells

| AML cells | HLA |
| --- | --- |
| THP-1 | HLA-A*0201 |
| OCI-AML2 | HLA-A*0201 |
| OCI-AML3 | HLA-A*0201 |
| MV4-11 | HLA-A*0301 |
| healthy donor 1 | HLA-A*0201 |
| healthy donor 2 | HLA-A*0301 |
