# Supplementary material for: CLPs-miR-103a-2-5p inhibits proliferation and promotes cell apoptosis in AML cells by targeting LILRB3 and Nrf2/HO-1 axis, regulating CD8 + T cell response
Source: J Transl Med. 2024 Mar 14;22:278. doi: 10.1186/s12967-024-05070-5 (PMC10938737; doi:10.1186/s12967-024-05070-5)
Supplement: Supplementary file 6 — Additional file 6. Four candidate miRNAs expression in AML cells. [file 12967_2024_5070_MOESM6_ESM.docx]

Fig. S1


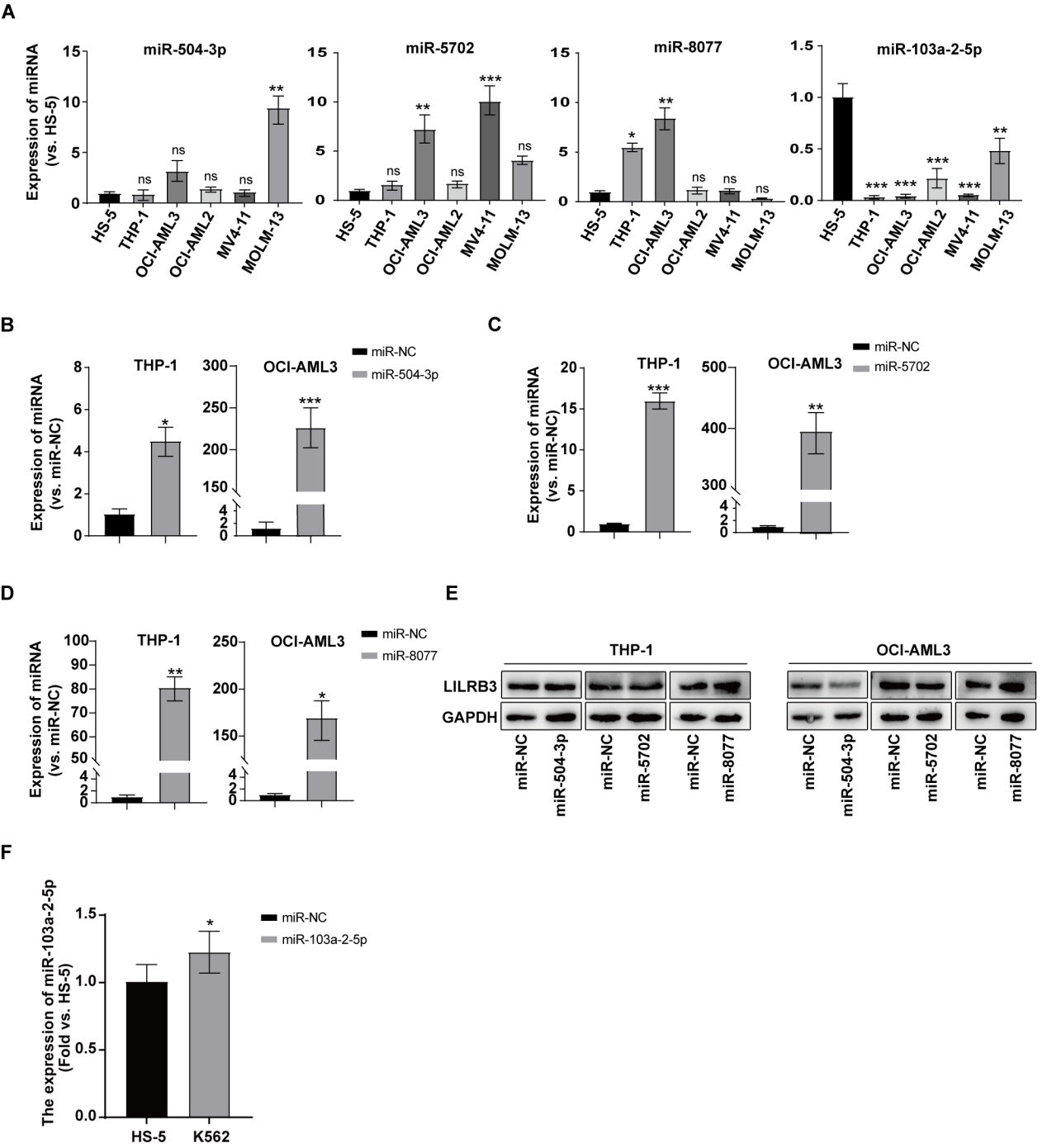


**Figure S1.** Four candidate miRNAs expression in AML cells.

(A) The level of miRNAs screened out by three algorithms in AML cell lines was detected by qRT-PCR. (B-D) The expression of miRNA in AML cells after transfection was detected by qRT-PCR. (E) The expression of LILRB3 at the protein levels was measured by western blotting analysis. (F) The expression of miR-103a-2-5p in K562 cells was detected by qRT-PCR. Multiple group comparison data were studied by One-Way ANOVA and Tukey’s post-hoc test. Statistical significance was determined as * P < 0.05, ** P < 0.01, *** P < 0.001, **** P < 0.0001, or no significant difference (ns) vs. HS-5 or miR-NC. Cell experiments were performed three times independently.
