# Supplementary material for: CLPs-miR-103a-2-5p inhibits proliferation and promotes cell apoptosis in AML cells by targeting LILRB3 and Nrf2/HO-1 axis, regulating CD8 + T cell response
Source: J Transl Med. 2024 Mar 14;22:278. doi: 10.1186/s12967-024-05070-5 (PMC10938737; doi:10.1186/s12967-024-05070-5)
Supplement: Supplementary file 7 — Additional file 7. The inhibitory effect of miR-103a-2-5p on the migration and colony formation of AML cells. [file 12967_2024_5070_MOESM7_ESM.docx]

Fig. S2


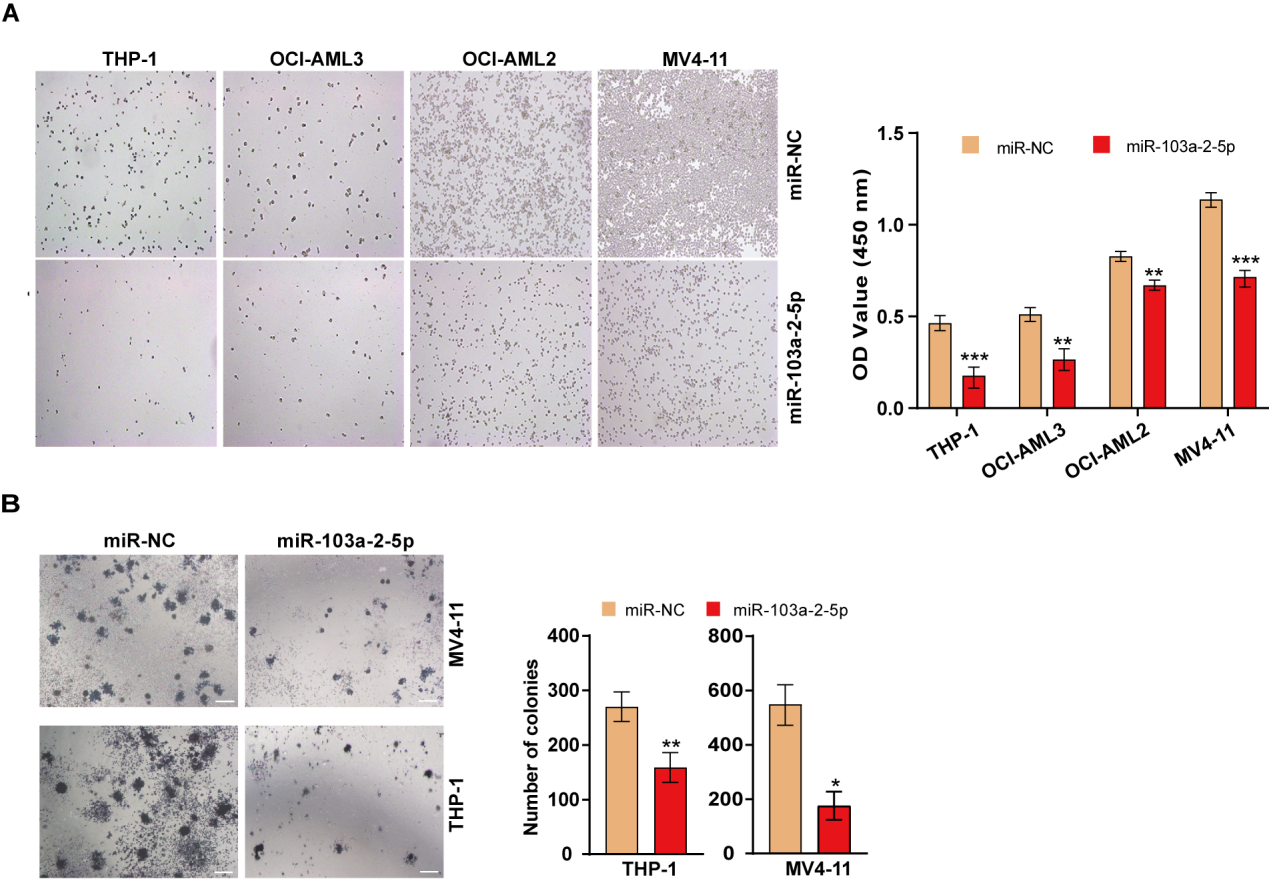


**Figure S2.** The inhibitory effect of miR-103a-2-5p on the migration and colony formation of AML cells.

(A) Transwell migration assays of THP-1, OCI-AML3, OCI-AML2, and MV4-11 cells transfected with miR-103a-2-5p or miR-NC. (B) Colony formation of THP-1 and MV4-11 cells transfecting with miR-103a-2-5p or miR-NC, cultured for 14 days. Statistical significance was determined as * P < 0.05, ** P < 0.01, *** P < 0.001 vs. control miRNA (miR-NC). Cell experiments were performed three times independently.
