# Supplementary material for: CLPs-miR-103a-2-5p inhibits proliferation and promotes cell apoptosis in AML cells by targeting LILRB3 and Nrf2/HO-1 axis, regulating CD8 + T cell response
Source: J Transl Med. 2024 Mar 14;22:278. doi: 10.1186/s12967-024-05070-5 (PMC10938737; doi:10.1186/s12967-024-05070-5)
Supplement: Supplementary file 8 — Additional file 8. MiR-103a-2-5p enhances CD8+ T cells survival and reduces inhibitory receptor generation. [file 12967_2024_5070_MOESM8_ESM.docx]

Fig. S3


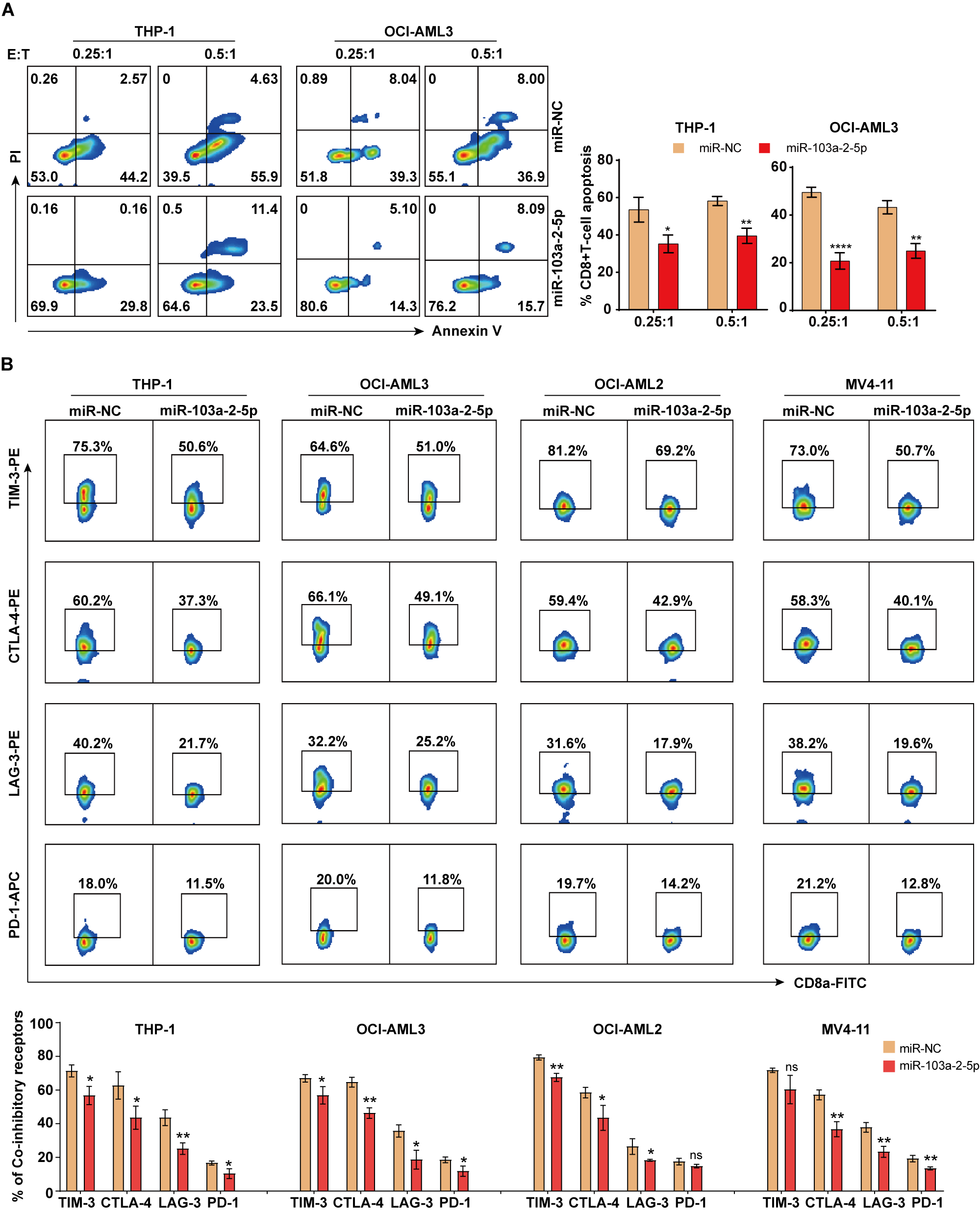


**Figure S3** MiR-103a-2-5p enhances CD8+ T cells survival and reduces inhibitory receptor generation. Tumor cells and PBMCs were co-cultured in the U-bottom 96-well plate for 72 h, and (A) CD8+ T cell apoptosis was detected by flow cytometry. (B) The expression of immune checkpoints on T cells was detected by flow cytometry. All results are presented as the mean ± SD, * P < 0.05, **P < 0.01, *** P < 0.001 vs. control miRNA (miR-NC). Cell experiments were performed three times independently.
