# Supplementary material for: CLPs-miR-103a-2-5p inhibits proliferation and promotes cell apoptosis in AML cells by targeting LILRB3 and Nrf2/HO-1 axis, regulating CD8 + T cell response
Source: J Transl Med. 2024 Mar 14;22:278. doi: 10.1186/s12967-024-05070-5 (PMC10938737; doi:10.1186/s12967-024-05070-5)
Supplement: Supplementary file 9 — Additional file 9. The mRNA expression of genes related to cell apoptosis, cell cycle, and antioxidation. [file 12967_2024_5070_MOESM9_ESM.docx]

Fig. S4


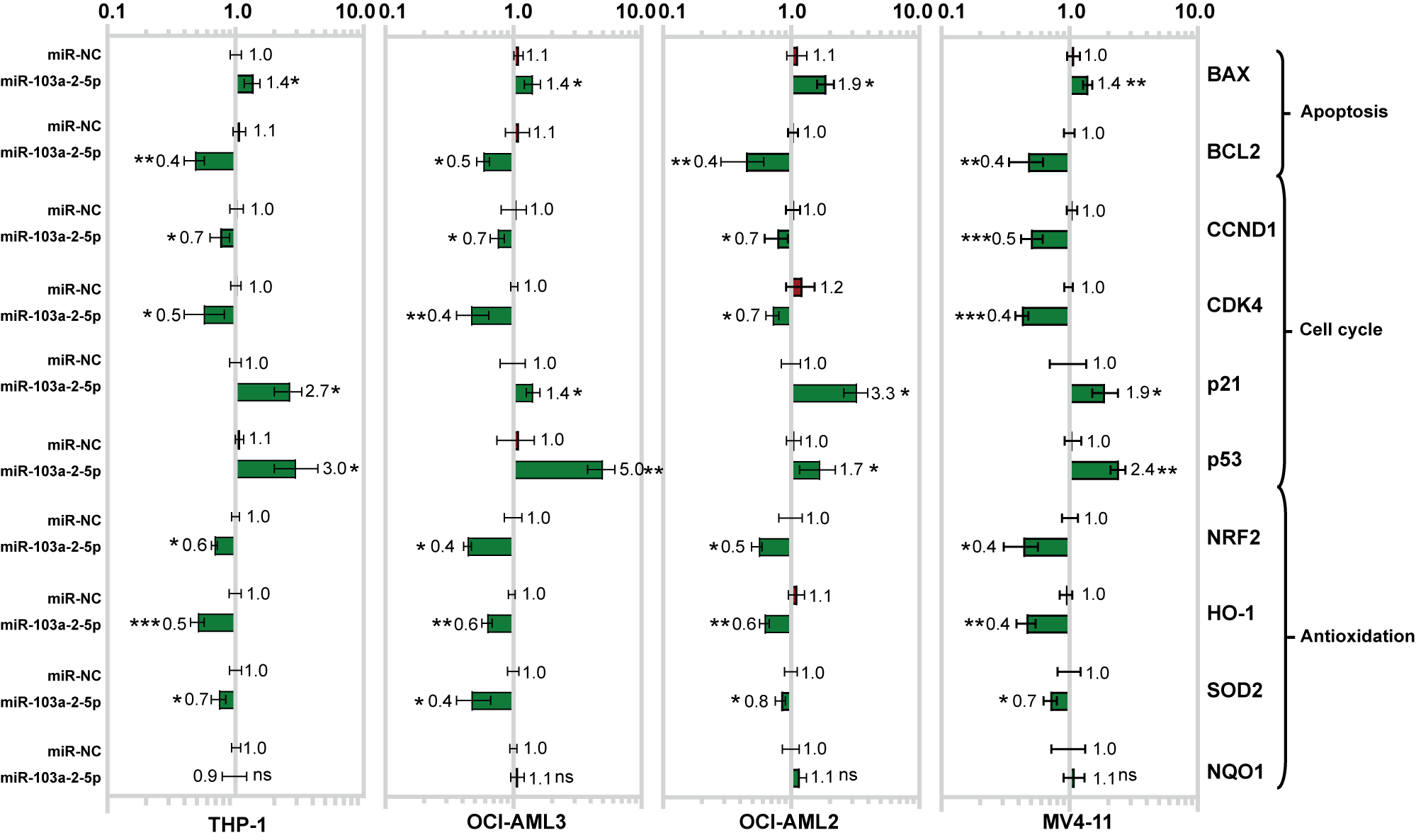


**Figure S4.** The mRNA expression of genes related to cell apoptosis, cell cycle, and antioxidation.

The mRNA expression of genes was detected by qRT-PCR after AML cells transfected with miR-103a-2-5p or miR-NC. All results are presented as the mean ± SD, * P < 0.05, **P < 0.01, *** P < 0.001 vs. control miRNA (miR-NC). Cell experiments were performed three times independently.
