# Supplementary material for: CLPs-miR-103a-2-5p inhibits proliferation and promotes cell apoptosis in AML cells by targeting LILRB3 and Nrf2/HO-1 axis, regulating CD8 + T cell response
Source: J Transl Med. 2024 Mar 14;22:278. doi: 10.1186/s12967-024-05070-5 (PMC10938737; doi:10.1186/s12967-024-05070-5)
Supplement: Supplementary file 10 — Additional file 10. The influence of miR-103a-2-5p in the AML tail vein mouse model. [file 12967_2024_5070_MOESM10_ESM.docx]

Fig. S5


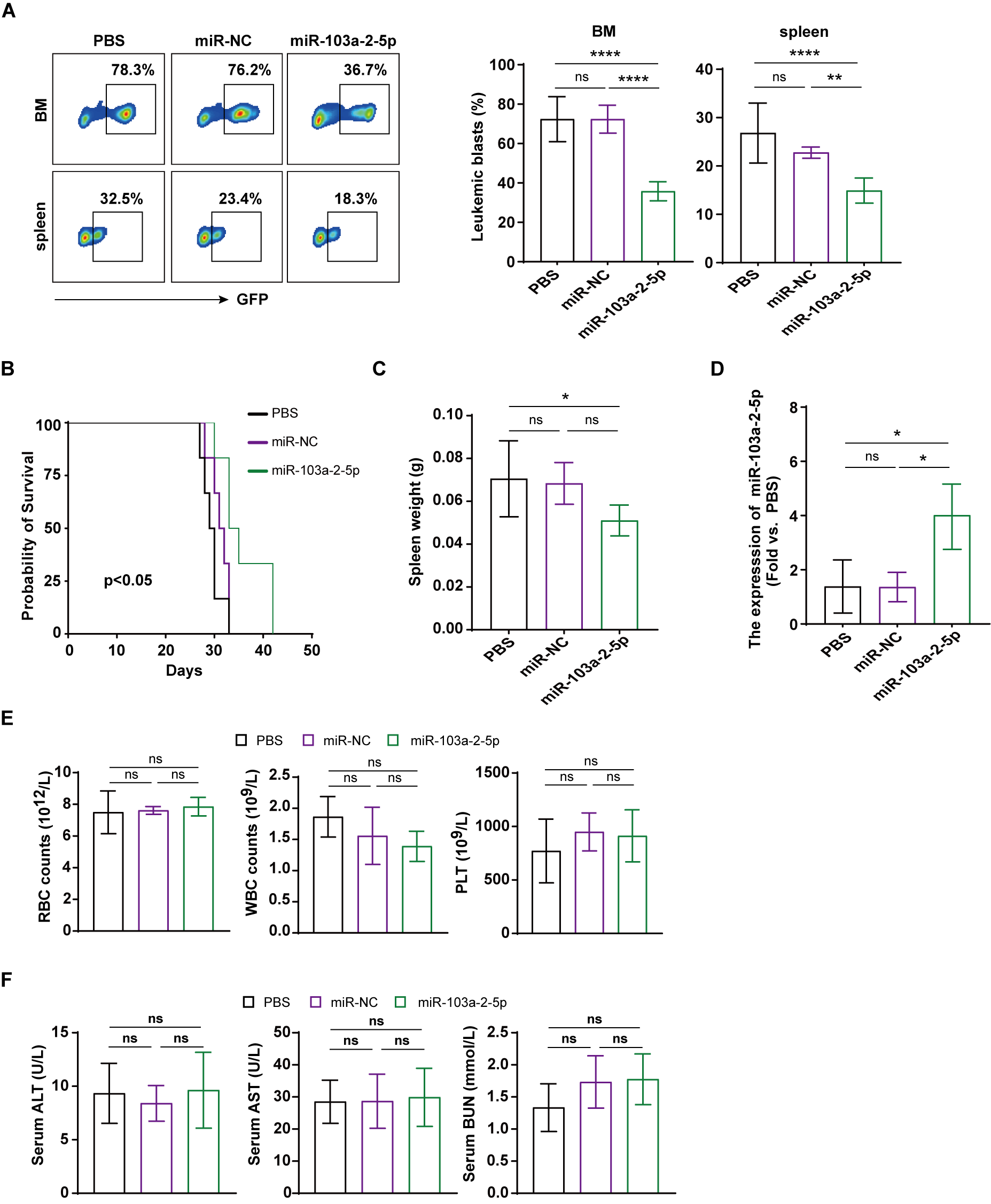


**Figure S5** The influence of miR-103a-2-5p in the AML tail vein mouse model.

(A) Flow cytometry analysis for detecting GFP-tagged THP-1 cells in the spleens and bone marrows of AML mice. (B) Animal survival curve. (C) The weight of spleens. (D) qRT-PCR for detecting the level of miR-103a-2-5p in mouse peripheral blood. (E) Routine blood test of mice after the treatment. (F) Changes in biochemical indicators of liver and kidney function in mice after the treatment. n=6; mean ± SD; * P < 0.05, **P < 0.01, *** P < 0.001, ns: no significance, vs. control miRNA (miR-NC) or PBS.
